# Nanodimensional and Nanocrystalline Apatites and Other Calcium Orthophosphates in Biomedical Engineering, Biology and Medicine

**DOI:** 10.3390/ma2041975

**Published:** 2009-11-27

**Authors:** Sergey V. Dorozhkin

**Affiliations:** Kudrinskaja sq. 1-155, Moscow 123242, Russia; E-Mail: sedorozhkin@yandex.ru; Tel.: +7-499-255-4460

**Keywords:** calcium orthophosphates, apatite, hydroxyapatite, nanocrystals, nanofibers, nanoparticles, nanopowders, nanostructured, nanodimensional, nanosized, biomaterials, bioceramics, biomineralization, tissue engineering, materials chemistry

## Abstract

Recent developments in biomineralization have already demonstrated that nanosized particles play an important role in the formation of hard tissues of animals. Namely, the basic inorganic building blocks of bones and teeth of mammals are nanodimensional and nanocrystalline calcium orthophosphates (in the form of apatites) of a biological origin. In mammals, tens to hundreds nanocrystals of a biological apatite were found to be combined into self-assembled structures under the control of various bioorganic matrixes. In addition, the structures of both dental enamel and bones could be mimicked by an oriented aggregation of nanosized calcium orthophosphates, determined by the biomolecules. The application and prospective use of nanodimensional and nanocrystalline calcium orthophosphates for a clinical repair of damaged bones and teeth are also known. For example, a greater viability and a better proliferation of various types of cells were detected on smaller crystals of calcium orthophosphates. Thus, the nanodimensional and nanocrystalline forms of calcium orthophosphates have a great potential to revolutionize the field of hard tissue engineering starting from bone repair and augmentation to the controlled drug delivery devices. This paper reviews current state of knowledge and recent developments of this subject starting from the synthesis and characterization to biomedical and clinical applications. More to the point, this review provides possible directions of future research and development.

## 1. Introduction

Living organisms can create the amazing ways to produce various high-performance materials and over 60 different inorganic minerals of biological origin have already been revealed [1]. Among them, calcium orthophosphates are of a special importance since they are the most important inorganic constituents of hard tissues in vertebrates [2,3]. In the form of a non-stoichiometric, ion-substituted and calcium deficient hydroxyapatite (commonly referred to as “biological apatite”), calcium orthophosphates are present in bones, teeth, deer antlers and tendons of mammals to give these organs stability, hardness and function [2,4,5]. Through we still do not exactly know why the highly intelligent animals use conformable calcium orthophosphates as their crucial biomineral for survival [6], current biomedical questions of persistent pathological and physiological mineralization in the body force people to focus on the processes, including the occurrence, formation and degradation of calcium orthophosphates in living organisms [7,8,9].

Biological mineralization (or biomineralization) is a process of *in vivo* formation of inorganic minerals [1,2]. In the biomineralization processes, organized assemblies of organic macromolecules regulate nucleation, growth, morphology and assembly of inorganic crystals. Biologically formed calcium orthophosphates (biological apatite) are always nanodimensional and nanocrystalline, which have been formed *in vivo* under mild conditions. According to many reports, dimensions of biological apatite in the calcified tissues always possess a range of a few to hundreds of nanometers with the smallest building blocks on the nanometer size scale [2,4,5,10,11]. For example, tens to hundreds of nanometer-sized apatite crystals in a collagen matrix are combined into self-assembled structures during bone and teeth formation [2,4,5]. Recent advances suggest that this is a natural selection, since the nanostructured materials provide a better capability for the specific interactions with proteins [12].

It is well established that nanodimensional and nanocrystalline forms of calcium orthophosphates can mimic both the composition and dimensions of constituent components of calcified tissues. Thus, they can be utilized in biomineralization and as biomaterials due to the excellent biocompatibility [13,14]. Further development of calcium orthophosphate-based biomaterials obviously will stand to benefit mostly from nanotechnology [15], which offers unique approaches to overcome shortcomings of many conventional materials. For example, nanosized ceramics can exhibit significant ductility before failure contributed by the grain-boundary phase. Namely, already in 1987, Karch *et al.* reported that, with nanograin dimensions, a brittle ceramic could permit a large plastic strain up to 100% [16]. In addition, nanostructured ceramics can be sintered at lower temperatures; thereby major problems associated with a high temperature sintering are also decreased. Thus, nanodimensional and nanocrystalline forms of bioceramics clearly represent a promising class of orthopedic and dental implant formulations with improved biological and biomechanical properties [17].

Many other advances have been made in biomaterial field due to a rapid growth of nanotechnology [18]. For example, a recent theory of “aggregation-based crystal growth” [19] and a new concept of “mesocrystals” [20,21] highlighted the roles of nanoparticles in biological crystal engineering. In this aspect, the study of calcium orthophosphates is a specific area in nanotechnology, because they might be applied readily to repair hard skeletal tissues of mammals [22,23,24].

Herein, an overview of nanodimensional and nanocrystalline apatites and other calcium orthophosphates in studies on biomineralization and biomaterials is given. To narrow the subject of the review, with a few important exceptions, undoped and un-substituted calcium orthophosphates are considered and discussed only. The readers interested in various nanodimensional and nanocrystalline ion-substituted calcium orthophosphates [25,26,27,28,29,30,31,32,33,34,35,36,37,38,39,40,41,42,43,44,45,46,47,48,49,50,51], calcium orthophosphate-based nanocomposites [52,53,54,55,56,57,58,59,60,61,62,63,64,65,66,67,68,69,70] or nanodimensional calcium orthophosphate-based composites [71,72,73,74,75,76,77,78,79,80,81,82,83,84,85,86] are advised to read the original papers. Furthermore, additional details on and more examples of calcium orthophosphate-based nanocomposites might be found in the chapter “Nano-calcium orthophosphate-based biocomposites and nano-biocomposites” in ref. [87].

This review is organized into several sections. After introduction (current section), general knowledge on calcium orthophosphates is provided in the second section. In the third section, general information on “nano” is discussed. The forth section briefly compares the micron-sized and nanodimensional calcium orthophosphates. The fifth section briefly discusses the presence of nanosized and nanocrystalline calcium orthophosphates in normal calcified tissues of mammals. The structure of nanosized and nanocrystalline apatites is described in the sixth section. Synthesis of nanodimensional and nanocrystalline calcium orthophosphates of various dimensions and shapes is reviewed in the seventh section, while the biomedical applications are examined in the eighth section. Finally, the summary and reasonable future perspectives in this active research area are given in the last section.

## 2. General Knowledge on Calcium Orthophosphates

The main driving force behind the use of calcium orthophosphates as bone substitute materials is their chemical similarity to the mineral component of mammalian bones and teeth [2,5,88,89]. As a result, in addition to being non-toxic, they are biocompatible, not recognized as foreign materials in the body and, most importantly, both exhibit bioactive behavior and integrate into living tissue by the same processes active in remodeling healthy bone. This leads to an intimate physicochemical bond between the implants and bone, termed osteointegration [90]. More to the point, calcium orthophosphates are also known to support osteoblast adhesion and proliferation [91,92]. Even so, the major limitations to use calcium orthophosphates as load-bearing biomaterials are their mechanical properties; namely, they are brittle with a poor fatigue resistance [93,94,95]. The poor mechanical behavior is even more evident for highly porous ceramics and scaffolds because porosity greater than 100 µm is considered as the requirement for proper vascularization and bone cell colonization [96,97,98]. That is why, in biomedical applications calcium orthophosphates are used primarily as fillers and coatings [88,89].

The complete list of known calcium orthophosphates, including their standard abbreviations and the major properties, is given in Table 1, while the detailed information on calcium orthophosphates, their synthesis, structure, chemistry, other properties and biomedical application has been comprehensively reviewed recently [88,89], where the interested readers are referred to. Even more thorough information on various calcium orthophosphates might be found in books and monographs [99,100,101,102,103,104,105].

**Table 1:** Existing calcium orthophosphates and their major properties [88,89].

Ca/P ionic ratio	Compound	Chemical formula	Solubility at 25 °C, – log(K_s_)	Solubility at 25 °C, g/L	pH stability range in aqueous solutions at 25 °C
0.5	Monocalcium phosphate monohydrate (MCPM)	Ca(H_2_PO_4_)_2_·H_2_O	1.14	~18	0.0–2.0
0.5	Monocalcium phosphate anhydrous (MCPA)	Ca(H_2_PO_4_)_2_	1.14	~17	^[c]^
1.0	Dicalcium phosphate dihydrate (DCPD), mineral brushite	CaHPO_4_·2H_2_O	6.59	~0.088	2.0–6.0
1.0	Dicalcium phosphate anhydrous (DCPA), mineral monetite	CaHPO_4_	6.90	~0.048	^[c]^
1.33	Octacalcium phosphate (OCP)	Ca_8_(HPO_4_)_2_(PO_4_)_4_·5H_2_O	96.6	~0.0081	5.5–7.0
1.5	α-Tricalcium phosphate (α-TCP)	α-Ca_3_(PO_4_)_2_	25.5	~0.0025	^[a]^
1.5	β-Tricalcium phosphate (β-TCP)	β-Ca_3_(PO_4_)_2_	28.9	~0.0005	^[a]^
1.2–2.2	Amorphous calcium phosphate (ACP)	Ca_x_H_y_(PO_4_)_z_·nH_2_O, n = 3–4.5; 15–20% H_2_O	^[b]^	^[b]^	~5–12 ^[d]^
1.5–1.67	Calcium-deficient hydroxyapatite (CDHA)^[e]^	Ca_10-*x*_(HPO_4_)*_x_*(PO_4_)_6-*x*_(OH)_2-*x*_^[f]^ (0 < *x* < 1)	~85.1	0.0094	6.5–9.5
1.67	Hydroxyapatite (HA)	Ca_10_(PO_4_)_6_(OH)_2_	116.8	~0.0003	9.5–12
1.67	Fluorapatite (FA)	Ca_10_(PO_4_)_6_F_2_	120.0	~0.0002	7–12
2.0	Tetracalcium phosphate (TTCP), mineral hilgenstockite	Ca_4_(PO_4_)_2_O	38–44	~0.0007	^[a]^

^[a]^ These compounds cannot be precipitated from aqueous solutions. ^[b]^ Cannot be measured precisely. However, the following values were found: 25.7 ± 0.1 (pH = 7.40), 29.9 ± 0.1 (pH = 6.00), 32.7 ± 0.1 (pH = 5.28). ^[c]^ Stable at temperatures above 100 °C. ^[d]^ Always metastable. ^[e]^ Occasionally, CDHA is named as precipitated HA. ^[f]^ In the case *x* = 1 (the boundary condition with Ca/P = 1.5), the chemical formula of CDHA looks as follows: Ca_9_(HPO_4_)(PO_4_)_5_(OH).

## 3. General Information on “Nano”

The prefix “nano” specifically means a measure of 10^-9^ units. Although it is widely accepted that the prefix “nano” specifically refers to 10^-9^ units, in the context of nanosized and nanocrystalline materials, the units should only be those of dimensions, rather than of any other unit of the scientific measurements. Besides, for practical purposes, it appears to be unrealistic to consider the prefix “nano” to solely and precisely refer to 10 [106]. Currently, there is a general agreement that the subject of nanoscience and nanotechnology started after the famous talk: “There’s plenty of room at the bottom” given by the Nobel Prize winner for Physics Prof. Richard P. Feynman on December 26, 1959 at the annual meeting of the American Physical Society held at California Institute of Technology. This well-known talk has been widely published in various media (e.g., [107]).

In a recent extensive discussion about a framework for definitions presented to the European Commission, the nanoscale has been defined as being of the order of 100 nm or less. Similarly, a nanomaterial [108] has been defined as “any form of a material that is composed of discrete functional parts, many of which have one or more dimensions of the order of 100 nm or less” [110]. Other definitions logically follow this approach such as: a nanocrystalline material is “a material that is comprised of many crystals, the majority of which have one or more dimensions of the order of 100 nm or less” (normally, with presence of neither the micron-sized crystals nor an intergranular amorphous phase) and a nanocomposite is a “multi-phase material in which the majority of the dispersed phase components have one or more dimensions of the order of 100 nm or less” [106]. Similarly, nanostructured materials are defined as the materials containing structural elements (e.g., clusters, crystallites or molecules) with dimensions in the 1 to 100 nm range [111], nanocoatings represent individual layers or multilayer surface coatings of 1–100 nm thick, nanopowders are extremely fine powders with an average particle size in the range of 1–100 nm and nanofibers are the fibers with a diameter within 1–100 nm [112,113]. It also has been proposed to extend the lower size limit to 0.1 nm [114], which would include all existing organic molecules, allowing chemists to rightly claim they have been working on nanotechnology for very many years [115]. According to their geometry, all nanomaterials can be divided into three major categories: equiaxed, one dimensional (or fibrous) and two dimensional (or lamellar) forms. Selected examples and typical applications of each category of nanomaterials and their use in biomedical applications are available in literature [116]. It is important to note, that in literature on calcium orthophosphates there are cases, when the prefix “nano” has been applied for the structures, with the minimum dimensions exceeding 100 nm [42,70,117,118,119,120,121,122,123].

As a rule, engineered nanomaterials can be manufactured from nearly any substance. Of crucial importance, there are two major characteristics conferring the special properties of any nanomaterial. These are the quantum effects associated with the very small dimensions (currently, this is not applicable to the biomaterials field) and a large surface-to-volume ratio that is encountered at these dimensions. For instance, specific surface areas for submicron-sized particles are typically 60–80 m^2^/g, while decreasing particle diameter to tens of nanometers increases the specific surface area up to 5 times more–an amazing amount of surface area per mass! Furthermore, all nanophase materials have the unique surface properties, such as an increased number of grain boundaries and defects on the surface, huge surface area and altered electronic structure, if compared to the micron-sized materials [106,124]. While less than ~1% of a microparticle’s atoms occupy the surface positions, over a tenth of the atoms in a 10-nm diameter particle reside on its surface and ~60% in a 2-nm particle [125]. This very high surface-to-volume ratio of nanomaterials provides a tremendous driving force for diffusion, especially at elevated temperatures, as well as causes a self-aggregation into larger particles. Besides, solubility of many substances increases with particle size decreasing [126,127]. What’s more, nanophase materials could have surface features (e.g., a higher amount of nanoscale pores) to influence the type and amount of adsorption of selective proteins that could enhance specific osteoblast adhesion [128]. Finally and yet importantly, the nanodimensional and nanocrystalline materials have different mechanical, electrical, magnetic and optical properties if compared to the larger grained materials of the same chemical composition [129,130,131,132].

The nanostructured materials can take the form of powders, dispersions, coatings or bulk materials. In general, nanostructured materials contain a large volume fraction (greater than 50%) of defects such as grain boundaries, interphase boundaries and dislocations, which strongly influences their chemical and physical properties. The great advantages of nanostructuring were first understood in electronic industry with the advent of thin film deposition processes. Other application areas have followed. For example, nanostructured bioceramics was found to improve friction and wear problems associated with joint replacement components because it was tougher and stronger than coarser-grained bioceramics [133]. Furthermore, nanostructuring has allowed chemical homogeneity and structural uniformity to an extent, which was once thought to impossible to achieve [111]. In calcium orthophosphate bioceramics, the major target of nanostructuring is to mimic the architecture of bones and teeth [134,135].

## 4. The Micron- and Submicron-Sized Calcium Orthophosphates versus Nanodimensional Ones

The micron-sized calcium orthophosphate-based bioceramic powders suffer from poor sinterability, mainly due to a low surface area (typically 2–5 m^2^/g), while the specific surface area of nanodimensional calcium orthophosphates exceeds 100 m^2^/g [136]. In addition, the resorption process of synthetic micron-sized calcium orthophosphates was found to be quite different from that of bone mineral [137].

Although the nanodimensional and nanocrystalline features of natural calcium orthophosphates of bones and teeth had been known much earlier [2,99,138,139,140,141,142], the history of the systematic investigations of this field has started only in 1994. Namely, a careful search in scientific databases using various combinations of keywords “nano” + “calcium phosphate”, “nano” + “apatite”, “nano” + “hydroxyapatite”, *etc*. in the article title revealed five papers published in 1994 [143,144,145,146,147]. No papers published before 1994 with the aforementioned keywords in the title were found.

Nanodimensional (size ~67 nm) HA was found to have a higher surface roughness of 17 nm if compared to 10 nm for the submicron-sized (~180 nm) HA, while the contact angles (a quantitative measure of the wetting of a solid by a liquid) were significantly lower for nanosized HA (6.1) if compared to the submicron-sized HA (11.51). Additionally, the diameter of individual pores in nanodimensional HA compacts is several times smaller (pore diameter ~6.6 Å) than that in the submicron grain-sized HA compacts (pore diameter within 19.8–31.0 Å) [148]. A surface roughness is known to enhance the osteoblast functions while a porous structure improves the osteoinduction compared with smooth surfaces and nonporpous structure, respectively [128]. Furthermore, nanophase HA appeared to have ~11% more proteins of fetal bovine serum adsorbed per 1 cm^2^ than submicron-sized HA [149]. Interfacial interactions between calcined HA nanocrystals and various substrates were studied and a bonding strength appeared to be influenced not only by the nature of functional groups on the substrate but also by matching of surface roughness between the nanocrystals and the substrate [150]. More to the point, incorporating of HA nanoparticles into polyacrylonitrile fibers were found to result in their crystallinity degree rising by about 5% [151].

In general, nanostructured biomaterials [152] offer much improved performances than their larger particle sized counterparts due to their huge surface-to-volume ratio and unusual chemical synergistic effects. Such nanostructured systems constitute a bridge between single molecules and bulk material systems [153]. For instance, powders of nanocrystalline apatites [154,155,156,157,158,159,160] and β-TCP [161] were found to exhibit an improved sinterability and enhanced densification due to a greater surface area. This is explained by the fact that the distances of material transport during the sintering becomes shorter for ultrafine powders with a high specific surface area, resulting in a densification at a low temperature. Therefore, due to low grain growth rates, a low-temperature sintering appears to be effective to produce fine-grained apatite bioceramics [162]. Furthermore, the mechanical properties (namely, hardness and toughness) of HA bioceramics appeared to increase as the grain size decreased from sub-micrometers to nanometers [163].

More to the point, nanosized HA is also expected to have a better bioactivity than coarser crystals [164,165,166]. Namely, Kim *et al.* found that osteoblasts (bone-forming cells) attached to the nanosized HA/gelatin biocomposites to a significantly higher degree than to micrometer size analog did [167]. An increased osteoblast and decreased fibroblast (fibrous tissue-forming cells) adhesion on nanophase ceramics [148,168,169,170,171,172], as well as on nanocrystalline HA coatings on titanium, if compared to traditionally used plasma-sprayed HA coatings, was also discovered by other researchers [173,174,175]. Scientists also observed enhanced osteoclast (bone-resorbing cells) functions to show healthy remodeling of bone at the simulated implant surface [165]. Besides, the proliferation and osteogenic differentiation of periodontal ligament cells were found to be promoted when a nanophase HA was used, if compared to dense HA bioceramics [176]. Thus, the underlying material property, responsible for this enhanced osteoblast function, is the surface roughness of the nanostructured surface [18]. Interestingly, but an increased osteoblast adhesion was discovered on nanoparticulate calcium orthophosphates with higher Ca/P ratios [177], which points out to some advantages of apatites over other calcium orthophosphates. Furthermore, a histological analysis revealed a superior biocompatibility and osteointegration of bone graft substitutes when nanosized HA was employed in biocomposites [178,179]. However, data are available that nanosized HA could inhibit growth of osteoblasts in a dose-dependent manner [180].

Obviously, the volume fraction of grain boundaries in nanodimensional calcium orthophosphates is increased significantly leading to improved osteoblast adhesion, proliferation and mineralization. Therefore, a nanocomposition of these biomaterials emulates the bone’s hierarchic organization, to initiate the growth of an apatite layer and to allow for the cellular and tissue response of bone remodeling. These examples emphasize that nanophase materials deserve more attention in improving orthopedic implant failure rates. However, to reduce surface energy, all nanosized materials tend to agglomerate and, to avoid the self-aggregation of calcium orthophosphate nanoparticles [181,182,183], special precautions might be necessary [120,184,185,186].

Finally yet importantly, CDHA nanocrystals obtained by precipitation methods in aqueous solutions were shown to exhibit physico-chemical characteristics rather similar to those of bone apatite [187]. In particular, their chemical composition departs from stoichiometry by calcium and hydroxide ions deficiency, leading to an increased solubility, and in turn bioresorption rate *in vivo* [88,89,99,100]. The CDHA nanocrystals have also a property to evolve in solution (maturation) like bone crystals. Namely, freshly precipitated CDHA has been shown to be analogous to embryonic bone mineral crystals whereas aged precipitates resemble bone crystals of old vertebrates [187].

## 5. Nanodimensional and Nanocrystalline Calcium Orthophosphates in Normal Calcified Tissues of Mammals

### 5.1. Bones

**Figure 1:**
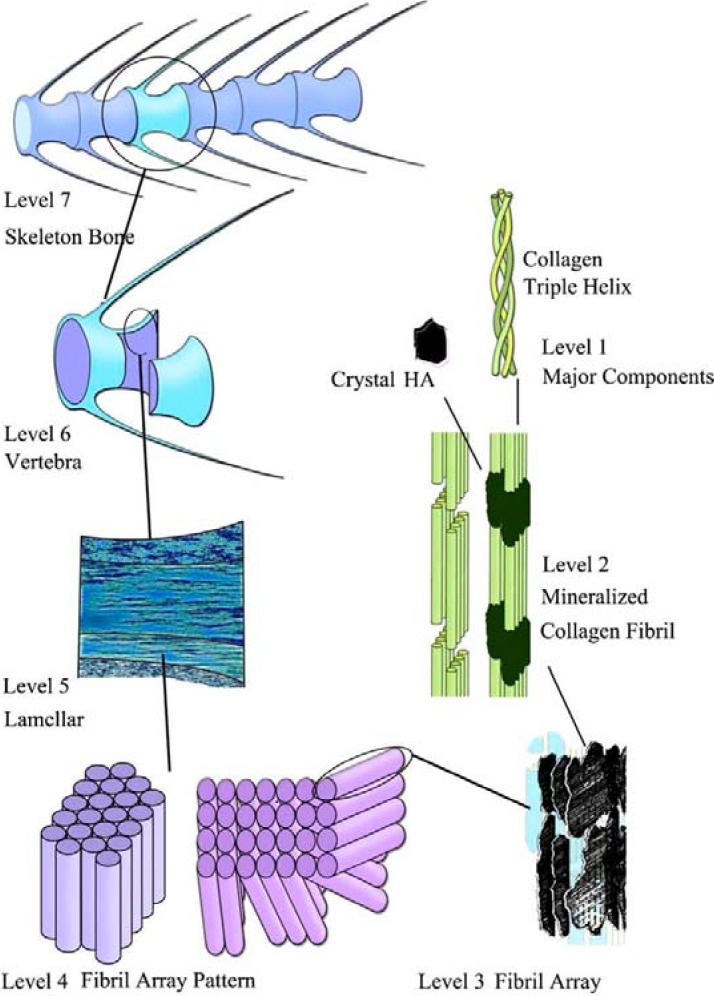
Seven hierarchical levels of organization of the zebra fish skeleton bone. Level 1: Isolated crystals and part of a collagen fibril with the triple helix structure. Level 2: Mineralized collagen fibrils. Level 3: The array of mineralized collagen fibrils with a cross-striation periodicity of nearly 60–70 nm. Level 4: Two fibril array patterns of organization as found in the zebra fish skeleton bone. Level 5: The lamellar structure in one vertebra. Level 6: A vertebra. Level 7: Skeleton bone. Reprinted from Ref. [189] with permission. Other good graphical sketches of the hierarchical structure of bones are available in Refs. [5,190].

Bone is the most typical calcified tissue of mammals and it comes in all sorts of shapes and sizes in order to achieve various functions of protection and mechanical support for the body. The major inorganic component of bone mineral is a biological apatite, which might be defined as a poorly crystalline (almost amorphous), non-stoichiometric and ion substituted CDHA [2,3,4,5,88,89,188]. From the material point of view, bone can be considered as an assembly of distinct levels of seven hierarchical structural units from macro- to micro- and to nanoscale (Figure 1) to meet numerous functions [2,5,124,189,190,191]. Furthermore, all these levels of bones permanently interact with cells and biological macromolecules. At the nanostructural level, tiny plate-like crystals of biological apatite in bone occur within the discrete spaces within the collagen fibrils and grow with specific crystalline orientation along the c-axes, which are roughly parallel to the long axes of the collagen fibrils [192]. Type I collagen molecules are self-assembled into fibrils with a periodicity of ~67 nm and ~40 nm gaps between the ends of their molecules, into which the apatite nanocrystals are placed. A composite of these two constituents forms mineralized fibers. The fibers also may be cross-linked, which provides a highly dynamic system capable of modification through the selection of different amino acids to allow for different mechanical properties for different biomaterial applications [193]. This is why bone is usually termed a fiber-reinforced composite of a biological origin, in which nanometer-sized hard inclusions are embedded into a soft protein matrix [194]. Though dimensions of biological apatite crystals reported in the literature vary due to different treatment methods and analytical techniques, it is generally around the nanometric level with values in the ranges of 30–50 nm (length), 15–30 nm (width) and 2–10 nm (thickness) [195]. Why does the nanometer scale appear to be so important to bones? It was recently demonstrated that natural nanocomposites exhibit a generic mechanical structure in which the nanometer sizes of mineral particles are used to ensure the optimum strength and maximum tolerance of flaws [196,197]. Furthermore, nanodimensional apatite has another crucial function for organisms. It is a huge reservoir of calcium and orthophosphate ions necessary for a wide variety of metabolic functions, which offer or consume calcium and orthophosphate ions through a so-called “remodeling” process because of a continuous resorption and formation of nanodimensional apatite by osteoclasts and osteoblasts, respectively, in a delicate equilibrium [2,5,88,89]. Further details on the bone structure, properties and composition might be found in literature [5,188,198].

### 5.2. Teeth

Teeth are another normal calcium orthophosphate-based calcified tissue of vertebrates. Unlike bone, teeth consist of at least two different biominerals: enamel (a crown, the part above the gum line) and dentin (root, the part below the gum line) [199]. Dental enamel contains up to 98% of biological apatite, ~1% of bioorganic compounds and up to 2% of water. Typical rods in enamel are composed of rod-like apatite crystals measuring 25–100 nm and an undetermined length of 100 nm to 100 μm or longer along the *c*-axis [200,201,202]. However, the apatite crystals in enamel were found to exhibit regular sub-domains or subunits with distinct chemical properties [203]. This subunit structure reflects an assembly mechanism for such biological crystals [204,205]. Like that for bones (Figure 1), seven levels of structural hierarchy have been also discovered in human enamel; moreover, the analysis of the enamel and bone hierarchical structures suggests similarities of the scale distribution at each level [206]. In enamel, nanocrystals of biological apatite at first form mineral nanofibrils; the nanofibrils always align lengthways, aggregating into fibrils and afterwards into thicker fibers; further, prism/interprism continua are formed from the fibers. At the microscale, prisms are assembled into prism bands, which present different arrangements across the thickness of the enamel layer. These compositional and structural characteristics endow enamel special properties such as anisotropic elastic modulus, effective viscoelastic properties, much higher fracture toughness and stress-strain relationships more similar to metals than ceramics [207].

Dentin contains ~50% of biological apatite, ~30% of bioorganic compounds and ~20% of water. In dentin, the nanodimensional building blocks (~25 nm width, ~4 nm thickness and ~35 nm length) of biological apatite are smaller than those of enamel. Dentin is analogous to bone in many aspects, for example, it has a similar composition and a hierarchical structure up to the level of the bone lamellae [88,89]. Further details on the structure and properties of teeth might be found elsewhere [208].

## 6. The Structure of the Nanodimensional and Nanocrystalline Apatites

Due to the apatitic structure on natural calcified tissues, apatites appear to be the best investigated compounds among the available calcium orthophosphates (Table 1). Thus, nanodimensional and nanocrystalline apatites have been extensively studied by various physico-chemical techniques and chemical analysis methods [183,209,210,211,212,213,214,215,216,217,218,219,220,221] with a special attention to the “nano” effect (*i.e.,* an enhanced contribution of the surface against the volume). Due to a nanocrystalline nature, various diffraction techniques have not yet given much information on the fine structural details related to apatite nanocrystals (assemblies of nanoparticles give only broad diffraction patterns, similar to ones from an amorphous material) [209,210]. Nevertheless, microdiffraction studies with electron microprobes 35 ± 10 nm in diameter clearly indicated a crystalline character of the nanoparticles in these assemblies. Furthermore, high-resolution transmission electron microscopy results revealed that HA nanoparticles behaved a fine monocrystalline grain structure [183,209].

**Figure 2:**
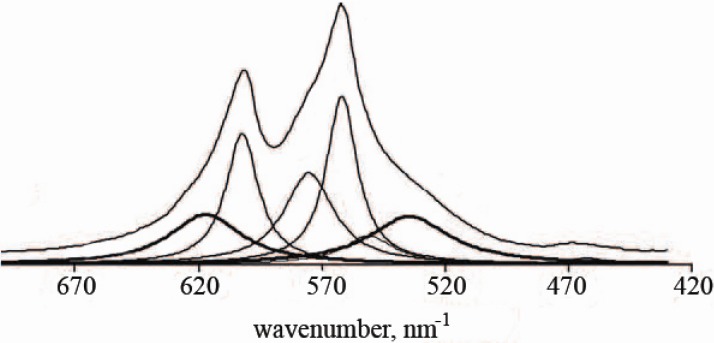
FTIR spectra of poorly crystalline apatites showing the non-apatitic environments of the orthophosphate ions (bold lines with peaks at 617 and 534 cm^-1^) and the apatitic PO_4_^3-^ (thin lines with peaks at 600, 575 and 560 cm^-1^) and HPO_4_^2-^ (thin line with peak at 550 cm^-1^) in the *ν*_4_ PO_4_ domain. Reprinted from Ref. [213] with permission.

Therefore, recent progress on the structure of nanodimensional and nanocrystalline apatites has relied mainly on diverse spectroscopic methods, which are sensitive to disturbances of the closest environments of various ions. Namely, the structure analysis revealed an existence of structural disorder at the particle surface, which was explained by chemical interactions between the orthophosphate groups and either adsorbed water molecules or hydroxyl groups located at the surface of apatite nanoparticles [211]. More to the point, infrared (FTIR) spectra of nanocrystalline apatites, in the *ν*_4_ PO_4_ domain, reveal the existence of additional bands of orthophosphate ions which cannot be assigned to an apatitic environment and which are not present in well-crystallized apatites (Figure 2). These bands have been assigned to non-apatitic environments of PO_4_^3-^ and HPO_4_^2-^ ions of the nanocrystals. Thus, FTIR spectra can be used to provide a sufficiently accurate evaluation of the amounts of such environments. Furthermore, the non-apatitic environments were found to correspond to hydrated domains of the apatite nanocrystals, which were distinct from the apatite domains [213]. Hence, precipitated apatite nanocrystals appeared to have a hydrated surface layer containing labile ionic species, which easily and rapidly can be exchanged with ions and/or macromolecules from the surrounding fluids [212,213,220]. For the as-precipitated apatites, such a layer appears to constitute mainly by water molecules coordinated to surface Ca^2+^ ions, approximately in the 1 : 1 ratio, while the OH groups account only for ~20% of the surface hydration species. The FTIR data indicated that water molecules, located on the surface of nanodimensional apatites, are coordinated to surface cations and experience hydrogen bonding significantly stronger than that in liquid water [219]. The surface hydrated layer is very delicate and becomes progressively transformed into a more stable apatitic lattice upon ageing in aqueous media. Furthermore, it irreversibly altered upon drying [213]. Outgassing at increasing temperatures up to ~300 °C resulted in a complete surface dehydration, accompanied by a decrease of the capability to re-adsorb water. Combination of these data with rehydration tests suggested that a significant part of surface Ca^2+^ ions, once dehydrated, could undergo a relaxation inward the surface, more irreversibly as the outgassing temperature increased [218].

In another study, elongated nanocrystals of CDHA with a typical diameter of about 10 nm and length of ~30–50 nm were synthesized and investigated by X-ray diffraction and nuclear magnetic resonance techniques. The CDHA nanocrystals were shown to consist of a crystalline core with the composition close to the stoichiometric HA and a disordered (amorphous) surface layer of 1–2 nm thick [217,218] with the composition close to DCPD [216]. Based on the total Ca/P ratio, on the one hand, and the crystal shape, on another hand, a thickness of the DCPD surface layer along the main crystal axis was estimated to be ~1 nm [216]. A similar structure of a crystalline core with the composition of the stoichiometric HA and a disordered (amorphous) surface layer was found by other researchers [222]; however, in yet another study devoted to nanodimensional carbonateapatites [223], the model of a crystalline core and an outer amorphous layer was not confirmed. Perhaps, this discrepancy could be explained by the presence of carbonates. A lack of hydroxide in nanodimensional apatites was detected; an extreme nanocrystallinity was found to place an upper bound on OH^-^ possible in apatites [224].

**Figure 3:**
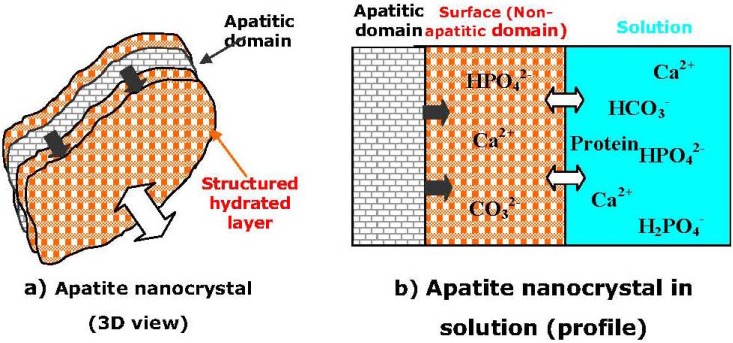
A schematic representation of the “surface hydrated layer model” for poorly crystalline apatite nanocrystals. Reprinted from Ref. [214] with permission.

After summarizing the available data, the following statements on the structure of apatite nanocrystals have been made: (1) they involve non-apatitic anionic and cationic chemical environments (in another study, the researchers mentioned on “ordered and disordered HA” [217]), (2) at least part of these environments are located on the surface of the nanocrystals and are in strong interaction with hydrated domains, (3) immature samples show FTIR band fine substructure that is altered upon drying without leading to long-range order modifications, (4) this fine substructure shows striking similarities with the FTIR spectrum of OCP [214]. All these elements favor a model in which apatite nanocrystals are covered with a rather fragile but structured surface hydrated layer containing relatively mobile ions (mainly, bivalent anions and cations: Ca^2+^, HPO_4_^2-^, CO_3_^2-^) in “non-apatitic” sites (Figure 3), which is supposed to be of either OCP or DCPD structure. Unfortunately, both the exact structure and the chemical composition of this hydrated layer are still uncertain (regrettably, as the hydrated layer cannot be isolated, it is not possible to standardize the methods for detailed studies) [214,216,217,218]. Nevertheless, it is known that the surface layer might adsorb considerable amounts of foreign compounds (molecules and ions) in the percent mass range [225]. Strictly speaking, all the aforementioned apply to both biological apatite of calcified tissues [226] and micron-sized apatites as well [227]; nonetheless, in nanocrystals, the composition of the hydrated surface layer contributes to the global composition for a non-negligible proportion. The results of electron states spectroscopy of nanostructural HA bioceramics are available elsewhere [228,229].

The hydrated surface layer confers unexpected properties to apatite nanocrystals, is responsible for most of the properties of apatites, and, for example, can help to explain the regulation by biological apatites of the concentration in mineral ions in body fluids (homeostasis). These properties are important for living organisms; therefore, they need to be used in both material science and biotechnology [213]. The consideration of this type of surface state can help understanding and explaining the behavior of biological apatites in participating in homeostasis due to a very high specific surface area of bone crystals and in constituting an important ion reservoir with an availability that depends on the maturation state. The important consequences are that the surface of nanodimensional apatites has nothing in common with the bulk composition and that the chemistry of such materials (e.g., binding of protein molecules) must be reconsidered [214,216]. Interestingly, but, in response to an electrical potential, the surface of HA nanobioceramics was found to exhibit dynamic changes in interfacial properties, such as wettability. The wettability modification enabled both a sharp switching from hydrophilic to hydrophobic states and a microscopic wettability patterning of the HA surface, which may be used for fabrication of spatially arrayed HA for biological cells immobilization or gene transfer [230].

Furthermore, a dry powder of nanodimensional HA was found to contain an X-ray amorphous portion with an unspecified location [231]. After mixing of an initial nanosized HA powder with a physiological solution (aqueous isotonic 0.9% NaCl solution for injections), this amorphous portion was fully converted into the crystalline phase of HA. The initial crystallite average size (approximately 35 nm) was enlarged by a factor of about 4 within the first 100 min after mixing the powder with the physiological solution and no more structural changes were detected during the following period [231]. In the light of the aforementioned studies, presumably, the discovered X-ray amorphous component of the initial powder was located on the surface of nanodimensional HA. To conclude this part, unfortunately, no information on the structure of other nanodimensional calcium orthophosphates has been found in literature.

## 7. Synthesis of the Nanodimensional and Nanocrystalline Calcium Orthophosphates

### 7.1. General Nanotechnological Approaches

The synthesis of nanoscale materials has received considerable attention and their novel properties can find numerous applications, for example, in the biomedical field. This has encouraged the invention of chemical, physical and biomimetic methods by which such nanomaterials can be obtained [124]. Generally, all approaches for preparation of nanodimensional and nanocrystalline materials can be categorized as “bottom-up” and “top-down” ones [132,232]. The bottom-up approach forms first nanodimensional or nanocrystalline elements and then assembles them into the final nanostructured material. An example is production of a nanopowder and its compaction into the final product (e.g., hot-pressed or sintered nanostructured ceramics). The top-down approach starts from a bulk material and then, via different dimension decreasing techniques, such as ball milling, leads to the formation of nanodimensional materials [124].

### 7.2. Nanodimensional and Nanocrystalline Apatites

**Figure 4:**
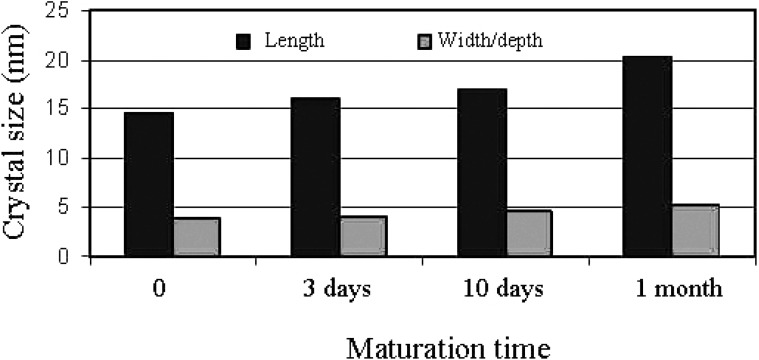
Variation of nanocrystalline apatite dimensions with maturation time. Reprinted from Ref. [214] with permission.

First of all, one should stress that the stoichiometric HA with well resolved X-ray diffraction patterns might be prepared mostly at temperatures exceeding ~700 °C either by calcining of CDHA with the Ca/P molar ratio very close to 1.67 or by solid-state reactions of other calcium orthophosphates with various chemicals (e.g., DCPA + CaO). Thus, with the exception of a hydrothermal synthesis [233,234,235], in aqueous solutions only CDHA might be prepared [88,89,99,100,101,102,103,104,105]. As all apatites (CDHA, HA and FA) belong to the sparingly soluble compounds (Table 1), simple mixing of calcium- and orthophosphate-containing aqueous solutions at pH > 9 results in formation of extremely supersaturated solutions and, therefore, a very fast precipitation of the tremendous amounts of very fine crystals [236], initially of ACP, that afterwards is re-crystallized into apatites [88,89,237,238,239,240]. The dimensions of the precipitated nanocrystals might be slightly increased by the Ostwald ripening approach (maturation), that is, by boiling and/or ambient aging in the mother liquid (Figure 4) [145,157,187,214,234,239,240,241,242,243,244]. Heat treatment of ACP might be applied as well [245]. Therefore, preparation of nanodimensional and/or nanocrystalline apatites is not a problem at all and has been known for many years [145,146,246,247,248]; however, prefix “nano” had not been used before 1994. On the contrary, with the exception of a thermally stable FA (thus, big crystals of FA might be produced by a melt-growth process [249,250]), manufacturing of big crystals of both CDHA and HA still is a challenge.

Many different methodologies have been proposed to prepare nanodimensional and/or nanocrystalline structures [251,252,253,254,255,256]. Prior to describing them, it is important to stress that in the vast majority of the available literature on apatites, the authors do not tell the difference between CDHA and HA. Therefore, getting through scientific papers, an attentive reader often finds statements, as: “Because natural bone is composed of both organic components (mainly type I collagen) and inorganic components (HA), …” [116], [116, p. 357], “The HA nanorods are synthesized via a wet precipitation process …” [155] [155, p. 2364], “… (TTCP) has been shown previously to be an essential component of self-setting calcium phosphate cements that form hydroxyapatite (HA) as the only end-product. …” [257] [257, abstract], *etc*. The matter with distinguishing between CDHA and HA becomes even much more complicated, when researchers deal with nanodimensional and/or nanocrystalline apatites because the assemblies of nanoparticles give only broad diffraction patterns, similar to ones from an amorphous material [209,210]. While composing this review, I always tried to specify whether each cited study dealt with CDHA or HA; unfortunately, the necessary data were found in just a few papers. Therefore, in many cases, I was forced to mention just “apatites” without a further clarification. Thus, the readers are requested to be understandable on this uncertainty.

To prepare nanodimensional and/or nanocrystalline apatites, methods of wet chemical precipitation [62,71,73,86,119,121,155,156,157,160,166,186,187,237,246,258,259,260,261,262,263,264,265,266,267,268,269,270,271,272,273,274,275,276,277,278,279,280,281,282,283,284,285,286,287,288], sol-gel synthesis [30,176,210,211,242,288,289,290,291,292,293,294,295,296,297,298,299], co-precipitation [243,300,301,302,303,304], hydrothermal synthesis [57,60,145,146,234,235,258,285,286,300,301,305,306,307,308,309,310,311,312,313,314,315,316,317,318,319], mechanochemical synthesis [53,228,312,317,320,321,322,323,324,325], mechanical alloying [326,327], ball milling [317,322,328,329], radio frequency induction plasma [330,331], vibro-milling of bones [332], flame spray pyrolysis [333], liquid-solid-solution synthesis [334], electrocrystallization [147,335,336], microwave processing [32,57,258,300,301,311,337,338,339,340,341,342,343,344], hydrolysis of other calcium orthophosphates [345,346,347], double step stirring [348], emulsion-based [274,349] or solvothermal [350] syntheses and several other techniques [31,43,137,143,247,351,352,353,354,355,356,357,358,359,360] are known. Continuous preparation procedures are also available [185,361]. Furthermore, nanodimensional HA might be manufactured by a laser-induced fragmentation of HA microparticles in water [362,363,364] and in solvent-containing aqueous solutions [297,313,365], while dense nanocrystalline HA films might be produced by radio frequency magnetron sputtering [366,367]. A comparison between the sol-gel synthesis and wet chemical precipitation technique was performed and both methods appeared to be suitable for synthesis of nanodimensional apatite [288].

**Figure 5:**
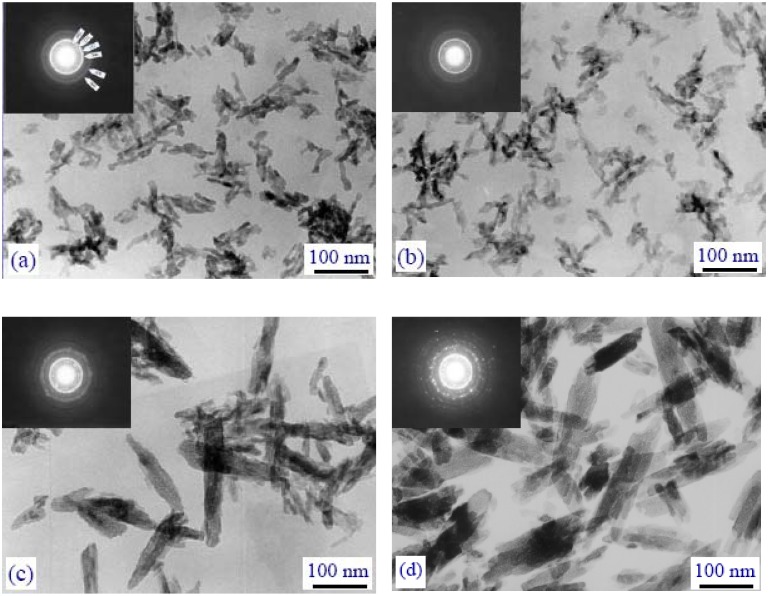
The influence of the reaction temperature on the crystal dimensions of precipitated CDHA: (a) –25 °C, (b) –37 °C, (c) –55 °C, (d) –75 °C.

Table 2 presents some data on the chronological development of synthesis of nanodimensional apatites for the period of 1995–2004 [137]. In general, the shape, stoichiometry, dimensions and specific surface area of the apatite nanoparticles appeared to be very sensitive to both the reaction temperature (Figure 5) and the reactant addition rate [270,279,285]. Furthermore, significant differences in the chemical composition, morphology and amorphous character of the CDHA nanoparticles produced through the reaction between aqueous solutions of Ca(NO_3_)_2_ and (NH_4_)_2_HPO_4_ can be induced, simply by changing the pH of the reactant hydrogen phosphate solution [374]. Among the methods described, the thinnest crystals of apatite (60 nm × 15 nm × 0.69 or 0.84 nm) have been prepared by Melikhov *et al.*; they have been called as “two dimensional crystalline HA” [266], while the smallest ones (size between 2.1 and 2.3 nm, *i.e.* around two times the HA unit cell parameters) have been found by Biggemann *et al.* [183]. Liu *et al.*, [375,376] and Han *et al.*, [373] synthesized nanosized HA via a template mediated and a non-template mediated sol-gel techniques, respectively. Both triethylphosphate [375,376] and other alkylphosphates [122] might be used to produce nanocrystalline apatites. Ion-substituted nanodimensional CDHA might be precipitated from both a synthetic [303] and a simulated [377] body fluids. A relatively simple sol-gel process using ethanol and/or water as a solvent has also been reported to obtain stoichiometric, nanocrystalline single phase HA [297].

Nanocrystalline HA powder was synthesized at a low calcination temperature of 750 °C by the citric acid sol-gel combustion method [373]. The attractive features of this method were to synthesize materials with a high purity, a better homogeneity and a high surface area in a single step [373,378]. An array of highly ordered HA nanotubes of uniform length and diameter was synthesized by sol-gel auto-combustion method with porous anodic aluminum oxide template [296]. Varma *et al.*, synthesized nanosized HA by polymeric combustion method and self-propagating combustion synthesis by using novel body fluid solutions [379]. Furthermore, nanoparticles of both FA and β-TCP might be synthesized by a simultaneous combustion of calcium carboxylate and tributylphosphate based precursors in a flame spray reactor [380]. Both a flame-based technique [381] and a spray drying approach [382] might be applied as well. Furthermore, crystalline and phase pure nanosized HA and CDHA were synthesized in a continuous hydrothermal flow system using supercritical water at t < 400 °C and 24 MPa pressure [307].

**Table 2:** Synthesis of nanodimensional apatites—a chronological development [137].

Year	Process	Reference
1995	Synthesis of nanocrystalline HA (particle size ~20 nm) for the first time using calcium nitrate and diammonium hydrogen orthophosphate as precursors by solution spray dry method.	[368]
2000	Synthesis of biomimetic nanosized CDHA powders (~50 nm) at 37 °C and pH of 7.4 from calcium nitrate tetrahydrate and diammonium hydrogen orthophosphate salts in synthetic body fluid using a novel chemical precipitation technique.	[303]
2002	Preparation of nanosized HA particles and HA/chitosan nanocomposite.	[369]
2002	Direct precipitation from dilute calcium chloride and sodium orthophosphate solutions.	[370]
2003	Radio frequency plasma spray process employing fine spray dried HA powders (average size ~15 μm) as a feedstock.	[330]
2003	Sol-gel process using equimolar solutions of calcium nitrate and diammonium hydrogen orthophosphate dissolved in ethanol.	[297]
2003	Chemical precipitation through aqueous solutions of calcium chloride and ammonium hydrogen orthophosphate.	[371]
2003	Dry mechanochemical synthesis of hydroxyapatites from dicalcium phosphate dihydrate and calcium oxide: a kinetic study.	[323]
2003	Synthesis of nano-powders via sucrose-templated sol-gel method using calcium nitrate and diammonium hydrogen orthophosphate as precursor chemicals.	[372]
2004	Hydrolysis method of DCPD and CaCO_3_ by 2.5 M NaOH (aq).	[345]
2004	Citric acid sol-gel combustion process using calcium nitrate tetrahydrate, diammonium hydrogen orthophosphate and citric acid.	[373]

Nanopowders of the stoichiometric HA of ~20 nm particle size were synthesized by hydrolysis of a mixture of DCPD and CaCO_3_ performed with 2.5 M aqueous solution of NaOH at 75 °C for 1 h. The only product synthesized was nanocrystalline HA and its crystallinity was improved with increasing annealing temperature [345]. Similar results were obtained in other studies [346,347]. Furthermore, Xu *et al.* used radio frequency plasma spray process to synthesize nanodimensional HA powders with particle size in the range of 10–100 nm [330]. Kuriakose *et al.* synthesized nanocrystalline HA of size ~1.3 nm that was thermally stable until 1200 °C [297]. Nanocrystalline plate-shaped particles of HA were directly precipitated at ambient temperature and pH ~7.4 from dilute aqueous solutions of calcium chloride and sodium orthophosphate. The direct precipitation of nanosized HA was achieved by submitting the aqueous suspension to microwave irradiation immediately after mixing [370]. A simple and easy approach for synthesizing thermally stable nanostructured stoichiometric HA powder under invariant pH conditions of 7.5, known as the NanoCaP process, was developed. Under these conditions, the synthesized HA not only remained in the nanostructured state but also did not exhibit any compositional fluctuations that were observed in conventional approaches for synthesizing HA [12]. Other examples of apatite nanoparticles preparation techniques might be found elsewhere [247]. Bulk bioceramics made of nanocrystalline HA with a grain size of no more than 50 nm and a near-theoretical density might be prepared by application of a high (~3.5 GPa) pressure in uniaxial compaction of nanopowders with subsequent sintering at 640 °C [156]. A similar approach has been reported by another research group [341].

Mechanochemical processing is another compelling method to produce nanostructured apatites in solid state [53,217,312,320,321,322,323,324]. For example, Yeong *et al.* used the appropriate amounts of DCPA and calcium oxide. The initial stage of mechanical activation resulted in a significant refinement in crystallite and particle sizes, together with a degree of amorphization in the starting powder mixture. This was followed by steady formation and subsequent growth of HA crystallites with increasing degree of mechanical activation. Finally, a single-phase HA of an average particle size of ~25 nm, a specific surface area of ~76 m^2^/g and a high crystallinity was attained after 20 h of mechanical activation [321]. A slightly different mechanism has been proposed in another study: during grinding, the acidic calcium phosphate (MCPM, DCPD or DCPA) reacted with a part of CaO (or Ca(OH)_2_), leading to ACP formation (rather than an amorphization of starting materials). Then the remaining CaO (or Ca(OH)_2_) reacted with ACP to give CDHA or HA according to the initial Ca/P ratio [323].

The use of macromolecules as templating agents to manipulate the growth of inorganic crystals has been realized in many biological systems. Namely, in the presence of biological macromolecules (such as collagen), nucleation and growth of nanocrystalline apatite to form highly organized bone minerals is one of the most fascinating processes in nature. They might be simulated. For example, layers of nanocrystalline apatite were formed *in situ* on the surface of various films at soaking them in aqueous solutions containing ions of calcium and orthophosphate. The *in situ* synthesized particles were found to be less agglomerated which was believed to be the result of nucleation of apatite crystallites on the regularly arranged side groups located on polymer chains [383,384]. Another approach comprises precipitation of nanodimensional apatites from aqueous solutions in the presence of dissolved high molecular weight polyacrylic acid [385,386] that acts as an inhibitor for the crystallization of apatitic crystals [387,388]. A similar inhibiting effect was found for dimethyl acetamide [389], polyvinyl alcohol [261] and several other (bio)polymers [390,391]. This type of synthesis is expected to lead to formation of nanocomposites, which might be structurally more comparable to bones with closely related mechanical and biological properties. Furthermore, a control of particle size of aqueous colloids of apatite nanoparticles was described involving a presence of amino acids [392,393]. The amino acids ensured effective growth inhibition by a predominant adsorption onto the Ca-rich surfaces during the initial stages of crystallization. Thus, the nanoparticles were formed by an oriented aggregation of primary crystallite domains along the *c*-axis direction. The size of the domains was shown to be governed by the interactions with the amino acid additives, which restricted a growth of the primary crystallites [392,393]. Furthermore, nanodimensional apatites might be precipitated from aqueous solutions of gelatin [58,394]. The development of apatite nanocrystals in aqueous gelatin solutions was highly influenced by the concentration of gelatin: namely, a higher concentration of gelatin induced formation of tiny (4 nm × 9 nm) nanocrystals, while a lower concentration of gelatin contributed to the development of bigger (30 nm × 70 nm) nanocrystals. In this experiment, a higher concentration of gelatin supplied abundant reaction sites containing groups such as carboxyl, which could bind with calcium ions. This lead to formation of a very large number of nuclei and creation of a large number of tiny nanocrystals [58].

Although each of the reported approaches to produce nanodimensional apatites has both a scientific and a practical relevance, a little attention has been dedicated to the physicochemical details involved in the careful control of the particle size distribution and particle shape. Indeed, in the case of particle size distribution, most of the reported ways to synthesize nanodimensional apatites really produced a particle mixture with a wide size distribution from tens to hundreds of nanometers. Moreover, the control of nanoparticle shape is another problem for these methods, which commonly result in pin-like or irregular particles. It is well known that bone consists of homogeneous plate-like nanocrystals of biological apatite of 15–30 nm wide and 30–50 nm long, while enamel consists of rod-like nanocrystals of biological apatite of 25–100 nm thick and lengths of 100 nm to microns [2,5,188,189,191,198,206,208]. The study of higher-level biomineralization and biomimetic assembly involves a search for advanced methods so that the synthesis of apatite nanocrystals can be accurately controlled [395]. For example, the size-controlled synthesis of materials can be achieved by using limited reaction spaces. Namely, microemulsions [318,396,397,398,399,400,401,402,403], micelles [404] and reverse micelles [308,405,406] have been successfully applied to synthesize nanodimensional apatites. In some cases, special polymers can be used as spatial reaction vessels for fabrication of CDHA. For example, Shchukin *et al.*, employed a poly(allylamine hydrochloride)/PO_4_^3-^ complex as a source of orthophosphate anions to capture calcium cations and make them react in the capsule volume [407]. Bose and Saha synthesized spherical-like nanocrystalline CDHA powder with particle diameters of ~30 and ~50 nm using the emulsion route [398]. Furthermore, nanocrystals of apatite might be aggregated into microspheres [408]. Hexadecyl (cetyl)trimethylammonium bromide (CTAB) was selected as an efficient agent to modulate the formation of CDHA nanoparticles [405,409]. The particle size can be regulated feasibly by changing the concentration of CTAB in the supersaturated by calcium orthophosphates solutions. For example, three different types of spherical CDHA nanoparticles with average diameters of 20 ± 5, 40 ± 10 and 80 ± 12 nm were fabricated using a series of CTAB concentrations to control the particle size. The experimental results revealed that the dimensions of the prepared CDHA nanoparticles were relatively uniform. In contrast, CDHA nanoparticles grown in the absence of organic additives are typical, rod-like particles with lengths of hundreds of nanometers and width of tens of nanometers [409].

To conclude this part, the surface of apatite nanoparticles might be functionalized by various compounds (even by quantum dots [410,411]) to provide new important properties [392,412,413,414,415,416,417], e.g., fluorescence [416] and luminescence [411,417].

### 7.3. Nanodimensional and Nanocrystalline TCP

Many researchers have formulated synthesis of nanodimensional β-TCP. For example, Bow *et al.*, synthesized β-TCP powders of ~50 nm particle diameter at room temperature in anhydrous methanol as a solvent [418]. With increase in aging time, the phase transformation was found to take place from initial DCPA, to intermediate ACP phases, then to final β-TCP. The authors observed that incorporation of carbonates helped in suppressing formation of ACP phases with apatitic structure and its transformation into poorly crystalline (almost amorphous) CDHA and favored the formation of β-TCP phase [418]. Nanoparticles of both FA and β-TCP were synthesized by a simultaneous combustion of calcium carboxylate and tributylphosphate based precursors in a flame spray reactor [380]. The same technique might be used to synthesize amorphous nanoparticles of unidentified TCP of 25–60 nm size [419,420,421,422], those after calcinations transformed into α-TCP or β-TCP. Nanodimensional β-TCP powders with an average grain size of ~100 nm [161,423] and less [424] were prepared by wet precipitation methods, followed by calcining at elevated temperatures. Both a sol-gel technique [425] and reverse micelle-mediated synthesis [426] are also applicable. In wet precipitation techniques, dialysis might be applied as a separation method [423]. When wet precipitation methods were used, initially nanodimensional CDHA with Ca/P ratio of ~1.50 was precipitated, that was transformed into nanosized β-TCP at calcination.

To synthesize TCP, both milling [427,428] and a high temperature flame spray pyrolysis [429] techniques might be employed as well. Afterwards, the nanodimensional β-TCP powders can be compacted into 3D specimens, followed by sintering to achieve the appropriate mechanical strength [161]. The maximal values of the bending strength, elastic modulus, Vickers hardness and compressive strength of the samples fabricated from nanosized β-TCP powders were more than two-times higher as compared to those of bioceramics obtained from microsized β-TCP powders. However, the degradability of bioceramics sintered from nanodimensional powders was just about one fourth of that sintered from microdimensional powders. Thus, the degradability of β-TCP bioceramics could be additionally regulated by the particle dimensions [161].

Nanowhiskers of several calcium orthophosphates (single-phase HA, single-phase β-TCP and biphasic HA + β-TCP) were produced by using a novel microwave-assisted “combustion synthesis (auto ignition)/molten salt synthesis” hybrid route. Aqueous solutions containing NaNO_3_, Ca(NO_3_)_2_ and KH_2_PO_4_ (with or without urea) were irradiated in a household microwave oven for 5 min at 600 watts of power. The as-synthesized precursors were then simply stirred in water at room temperature for 1 h to obtain the nanowhiskers of the desired calcium orthophosphate bioceramics [430]. Furthermore, nanostructured biphasic (HA + β-TCP) bioceramics was successfully prepared by microwave synthesis [431,432] and a polymer matrix mediated process [433] in other studies. Good cellular activities of the biphasic bioceramics have been reported.

Layrolle and Lebugle developed a synthesis route of nanosized FA and other calcium orthophosphates, using calcium diethoxide Ca(OEt)_2_) and H_3_PO_4_ [143] (+ NH_4_F to prepare FA [434]) as the initial reagents and anhydrous ethanol as a solvent. By a simple variance of the ratio of reagents, calcium orthophosphates of various chemical compositions were precipitated in ethanol. The precipitates were characterized and the results indicated that those calcium orthophosphates were amorphous and nanodimensional. Furthermore, they had large specific surface areas and possessed a high reactivity [143,434].

### 7.4. Other Nanodimensional and Nanocrystalline Calcium Orthophosphates

Nanoparticles of DCPD (with some amount of CDHA and ACP) of a relatively high monodispersity could be synthesized from aqueous solutions of calcium nitrate and orthophosphoric acid in the presence of 2-carboxyethylphosphonic acid. They are produced in a discoid shape with a diameter of 30–80 nm and a height of less than ~5 nm. They form stable colloidal solutions displaying minimal agglomeration [435]. An interesting approach comprises precipitation of calcium orthophosphates inside nano-sized pores of another material. For example, DCPD nanoclusters were immobilized into pores of an oxide network by immersion of this network into an acidic (pH = 2.7) calcium orthophosphate solution at 50 °C [436]. The acid-base reaction between the calcium orthophosphate solution and the hydroxyl groups of the oxide network resulted in formation of nanoclusters of DCPD immobilized inside the oxide pores. Interestingly, but the immobilized nanoclusters of DCPD were further converted into those of ACP and CDHA by supplementary treatment of the oxide network in alkaline solutions [436]. Hollow nanoshells of undisclosed calcium orthophosphates (presumably, of ACP) with a size distribution of (120–185) ± 50 nm and predictable mean shell thickness from 10 to 40 nm were prepared by crystallization onto the surface of nanodimensional liposomes [437,438]. Both the suspension stability and shell thickness control were achieved through the introduction of carboxyethylphosphoric acid. Variation of shell thickness and stoichiometry may be a way of manipulating the dissolution kinetics of ACP coating to control the release of encapsulated materials, necessary for drug delivery purposes [437,438]. Roughly spherical DCPA nanoparticles of approx. 50–100 nm in sizes were synthesized via a spray-drying technique [439,440,441], while ribbon-like fibers of DCPA might be prepared upon hydrolysis in urea [346]. Furthermore, nanodimensional calcium orthophosphate powders with DCPD as the major phase have been synthesized by an inverse microemulsion system using kerosene as the oil phase, a cationic surfactant and a non-ionic surfactant [442]. Microskeletal constructions might be synthesized as well [443].

When it comes to ACP, it is nanodimensional in the vast majority cases [104]. Approximately spherical ACP nanoparticles with a diameter of about 50 nm can be prepared by rapid precipitation from water and subsequent colloidal stabilization by coating with polymers [444]. Nanoclusters of ACP [445] or those comprising a spherical core of 355 ± 20 DCPD units with density of 2.31 g/cm^3^ and radius of 2.30 ± 0.05 nm surrounded by 49 ± 4 peptide chains with a partial specific volume of 0.7 cm^3^/g, forming a tightly packed shell with an outer radius of 4.04 ± 0.15 nm were prepared by precipitation using 10 mg/mL of the 25-amino-acid *N*-terminal tryptic phosphopeptide of bovine β-casein as a stabilizing agent [446]. Nanoparticles of ACP were prepared by mixing of solutions of Ca(NO_3_)_2_·4H_2_O (450 mmol/L) in acetone and (NH_4_)_2_HPO_4_ (30 mmol/L) in deionized water at pH within 10.0–11.0 [447]. Furthermore, nanopowders of ACP might be prepared by an electrostatic spray pyrolysis technique [448,449].

Self-assembled shell cross-linked poly(acrylic acid-b-isoprene) micelles and/or cross-linked poly(acrylic) acid nanocages in aqueous solutions might be used as templates for preparation of polymer/calcium orthophosphate nanocapsules with hybrid nanostructures of 50–70 nm in diameter, which consisted of spherical polymer nanoparticles or nanocages enclosed within a continuous 10–20 nm thick surface layer of ACP [450]. Synthesis of hollow spherical calcium orthophosphate nanoparticles using polymeric nanotemplates has been also reported by other researchers [451]. Furthermore, bundles of surfactant-coated ACP nanofilaments, ~2 nm in width and >300 μm in length were synthesized in reverse micelles [452]. The nanofilament bundles were found to be stable in the reverse micelle phase up to around 5 days, after which they transformed into 5 nm-wide surfactant-coated CDHA nanorods. Discrete nanofilaments (100–500 × 10–15 nm in size) consisting of a linear superstructure based on the side-on stacking of surfactant-coated ACP nanorods were also prepared [387]. A double reverse-micelle strategy was realized to synthesize amine, carboxylate- and polyethylene glycol surface functionalized calcium orthophosphate nanoparticles of an undisclosed nature [453]. Furthermore, the reverse micelle technique might be applied to prepare DCPA nanoparticles [405,454].

Pulsed laser deposition technique was employed to obtain thin films of nanocrystalline OCP on pure Ti substrates [455]. The deposition was performed by a pulsed UV laser source in a flux of hot water vapors. High-resolution electron microscopy and X-ray diffraction at grazing incidence investigations indicated that the coatings were made of nanocrystalline OCP (unfortunately, the dimensions were not indicated). *In vitro* tests proved that both fibroblasts and osteoblasts adhered, reached a normal morphology, proliferated and remained viable when cultured on the nanocrystalline OCP coatings, supporting a good biocompatibility and absence of any toxicity [455].

Similar to that for apatites (see above), the surface of both TCP and other calcium orthophosphate nanoparticles also might be functionalized by various compounds to provide new important properties [185,453,456,457,458,459,460,461], e.g., fluorescence [458,459] or a good disperseability in organic solvents [461]. Furthermore, calcium orthophosphate nanoparticles might be used as templates to manufacture nanocapsules [462].

### 7.5. Biomimetic Construction Using Calcium Orthophosphate Nanoparticles

Morphological control of bioinorganic materials is another interested issue in biomineralization, by which inorganic materials with complex morphologies can be produced. Complex forms or patterns with a hierarchical structure over several length scales are important features of biomineralization. Pattern formation in biomineralization is a process in which self-assembled organic templates are transformed by a material’s replication into organized inorganic structures. Needless to mention, that researchers try to reproduce these processes in laboratories. For example, Chen *et al.*, reported a way to create enamel-like structures by modifying synthetic apatite nanorods with a surfactant, bis(2-ethylhexyl)sulfosuccinate salt, that allowed the nanorods to self-assemble into prism-like structures at the water/air interface [204]. A nanometer-scale rod array of apatite having preferred orientation to the *c*-axis was successfully prepared simply by soaking calcium-containing silicate glass substrates in Na_2_HPO_4_ aqueous solution at 80 °C for various periods [463]. A biomimetic bottom-up route to obtain the first hierarchical level of bone was reported [193]. A pH-induced self-assembly of peptide-amphiphile to make a nanostructured fibrous scaffold reminiscent of extracellular bone matrix was obtained. After the cross-linking of the scaffold, the fibers were able to direct mineralization of CDHA to form a composite material, in which the crystallographic *c*-axes of the CDHA nanocrystals were aligned with the long axes of the fibers. This alignment was similar to that observed between collagen fibrils and crystals of biological apatite in bones [193]. Other attempts to fabricate artificial materials having bone-like nanostructure and chemical composition were performed and several significant achievements were obtained [464,465].

The classical model of biomineralization considers mineral formation as an amplification process in which individual atoms or molecules are added to existing nuclei or templates [1,2,466]. This process occurs in the presence of various bioorganic molecules, which deterministically modify nucleation, growth and facet stability. A model involving aggregation-based growth [467] recently challenged this conventional concept for the crystal growth. Inorganic nanocrystals were found to aggregate into ordered solid phases via oriented attachment to control the reactivity of nanophase materials in nature [19,468]. A new model of “bricks and mortar” was suggested to explain the biological aggregation of apatite nanoparticles [469]. In this model, ACP acts as “mortar” to cement the crystallized “bricks” of nanosized HA. Meanwhile, biological molecules control the nanoconstruction process. By using HA nanospheres as the building blocks, highly ordered enamel-like and bone-like apatites were hierarchically constructed in the presence of glycine and glutamate, respectively. It is interesting that, during the evolution of biological apatite, the amorphous “mortar” can be eventually turned into the “brick” by phase-to-phase transformation to ensure the integrity of biominerals [469].

## 8. Biomedical Applications of the Nanodimensional and Nanocrystalline Calcium Orthophosphates

### 8.1. Bone Repair

Due to advances in surgical practice and a fast aging of the population, there is a permanently increasing demand for bone grafts [470]. Modern grafts should not only replace the missing bones, but also should be intrinsically osteoinductive by acting as scaffolds for guided bone growth. Furthermore, an ability to form a biologically active apatite layer to bond to living bone it is an essential requirement to modern biomaterials [471]. In addition, a good graft should provide a framework to support new blood vessels and soft tissues in forming a bridge to existing bones [470].

**Figure 6:**
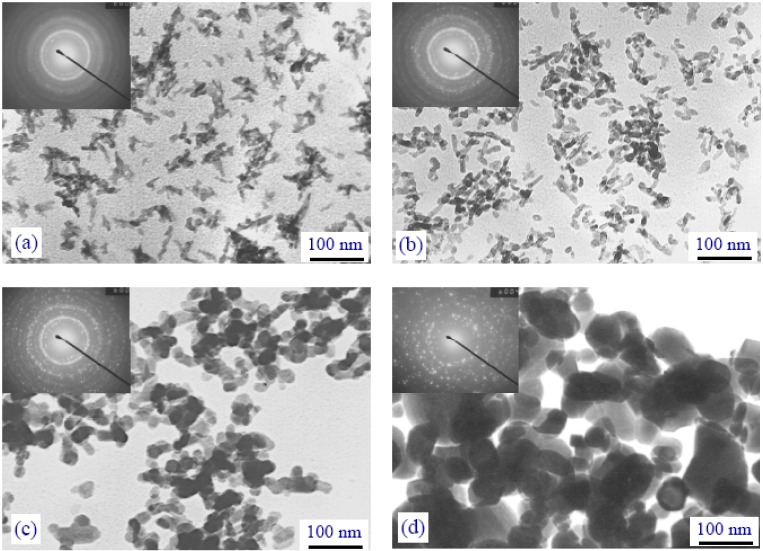
Particle sizes and crystallinity of HA powders after a heat treatment at various temperatures: (a) –300 °C, (b) –500 °C, (c) –700 °C, (d) –900 °C.

Calcium orthophosphate bioceramics of micron dimensions have been used in dentistry, orthopedics and surgery for over 30 years because of their chemical similarity to calcified tissues of mammals and, therefore, excellent biocompatibility [88,89,99,100,101,102]. Due to a rapid development of nanotechnology, the potential of nanodimensional and nanocrystalline calcium orthophosphates has received a considerable attention [18] because they produce favorable results in repair of bone defects [472]. For example, due to an improved sinterability, an enhanced densification and a better bioactivity than coarser crystals, they might be chosen as the major components of self-setting bone cements [14,25,419,420,473,474,475,476,477]. However, there is a study in which an increase of particle and crystallite sizes of TCP did not prolong but shortened the induction time until the cement setting reaction started [422], which was against the common physical rules (generally, smaller particles or crystallites should enhance reactivity). Nevertheless, two general directions of the biomedical application of nanodimensional and nanocrystalline calcium orthophosphates can be outlined: (i) using them in powder form as filling materials to impart bioactivity to various biocomposites and hybrid biomaterials [52,53,54,55,56,57,58,59,60,61,62,63,64,65,66,67,68,69,70,71,72,73,74,75,76,77,78,79,80,81,82,83,84,85,86,167,478]; (ii) manufacturing of either dense compacts or porous scaffolds, possessing the sufficient mechanical properties [62,79,264,265,464,465,479,480]. As the nanodimensional and nanocrystalline calcium orthophosphates tend to agglomerate at heating (Figure 6) [277,481,482,483], normally a low-temperature [157,297] and/or a rapid consolidation [157,235,286,484,485,486,487] techniques must be employed. The low-temperature approach comprises gel hardening (at 4 °C) [297] and uni-axial pressing at 150–200 °C [157]. The rapid consolidation techniques comprise spark plasma sintering [157,235,286,484] and microwave sintering over the temperature range 1000–1300 °C, using a rapid sintering schedule [485,486,487]. Furthermore, nanodimensional crystals of calcined HA might be fabricated by calcination at 800 °C for 1 h with an anti-sintering agent surrounding the original nanosized CDHA particles and the agent is subsequently removed by washing after the calcination [488,489,490]. These consolidation approaches provided a limited alteration of the initial nanocrystals, while the final bioceramics possessed the mechanical properties similar to those reached with sintered stoichiometric HA.

Already in 1990-s, implants prepared from nanodimensional apatites, as well as biocomposites of nanodimensional apatite with organic compounds were tested *in vivo* [491,492,493]. Cylinders made of both pure nanodimensional apatite and organoapatite containing a synthetic peptide were analyzed 28 days after implantation into spongy bones of Chinchilla rabbits. Both implant types were well incorporated and interface events were found to be similar to those observed on human bone surfaces with regard to resorption by osteoclast-like cells and bone formation by osteoblasts. That study revealed a suitability of such materials for both bone replacement and drug release purposes [491]. Similar results were obtained in other studies [492,493].

Among the available commercial formulations, NanOss^™^ bone void filler from Angstrom Medica, Inc. [494] is considered as the first nanotechnological medical device received the clearance by the US Food and Drug Administration (FDA) in 2005. It is prepared by precipitation of calcium orthophosphate nanoparticles from aqueous solutions and the resulting white powder is then compressed and heated to form a dense, transparent and nanocrystalline material. NanOss^™^ mimics the microstructure, composition and performance of human bone, as well as it is mechanically strong and osteoconductive. It is remodeled over time into human bone with applications in the sports medicine, trauma, spine and general orthopedics markets [494].

Ostim^®^ (Osartis GmbH & Co. KG, Obernburg, Germany) is another popular commercial formulation. It is a ready-to-use injectable paste that received CE (Conformite Europeenne) approval in 2002. Ostim^®^ is a suspension of synthetic nanocrystalline HA (average crystal dimensions: 100 × 20 × 3 nm^3^ (a needle-like appearance); specific surface area ~100 m^2^/g) in water, prepared by a wet chemical reaction [495]. After completion, the HA content in the paste is ~35%. Ostim^®^ does not harden when mixed with blood or spongiosa, so it is highly suitable for increasing the volume of autologous or homologous material. Simultaneously, its viscosity enables it to be applied to form-fit in close contact with the bone. Ostim^®^ can be used in metaphyseal fractures and cysts, alveolar ridge augmentation, acetabulum reconstruction and periprosthetic fractures during hip prosthesis exchange operations, osteotomies, filling cages in spinal column surgery, *etc*. [494,496,497,498,499,500,501,502,503,504,505,506]. It might be incorporated into bones and a new bone formation is visible after only three months [507]. For a number of clinical applications, Ostim^®^ might be combined with other types of calcium orthophosphate bioceramics, e.g., with a HA bioceramic core (Cerabone^®^) [495,508] or with biphasic (β-TCP + HA) granules (BoneSaves^®^) [509]. Application of such combinations of a nanocrystalline Ostim^®^ with the microcrystalline calcium orthophosphate bioceramics appeared to be an effective method for treatment of both tibia head compression fractures [495] and metaphyseal osseous volume defects in the metaphyseal spongiosa [508]. Besides, such combinations might be used for acetabular bone impaction grafting procedures [509].

Cui *et al.* developed nanosized HA/collagen biocomposites, which mimicked the nanostructure of bones [189,510]. After implantation, such biocomposites can be incorporated into bone metabolism. Due to processing difficulties and poor mechanical properties of bulk calcium orthophosphates, their applications are currently confined to non-load-bearing implants and porous bodies/scaffolds. Porous 3D nanocomposites of HA and collagen/polymer mimic bones in composition and microstructure and can be employed as a matrix for the tissue engineering of bone [74].

**Figure 7:**
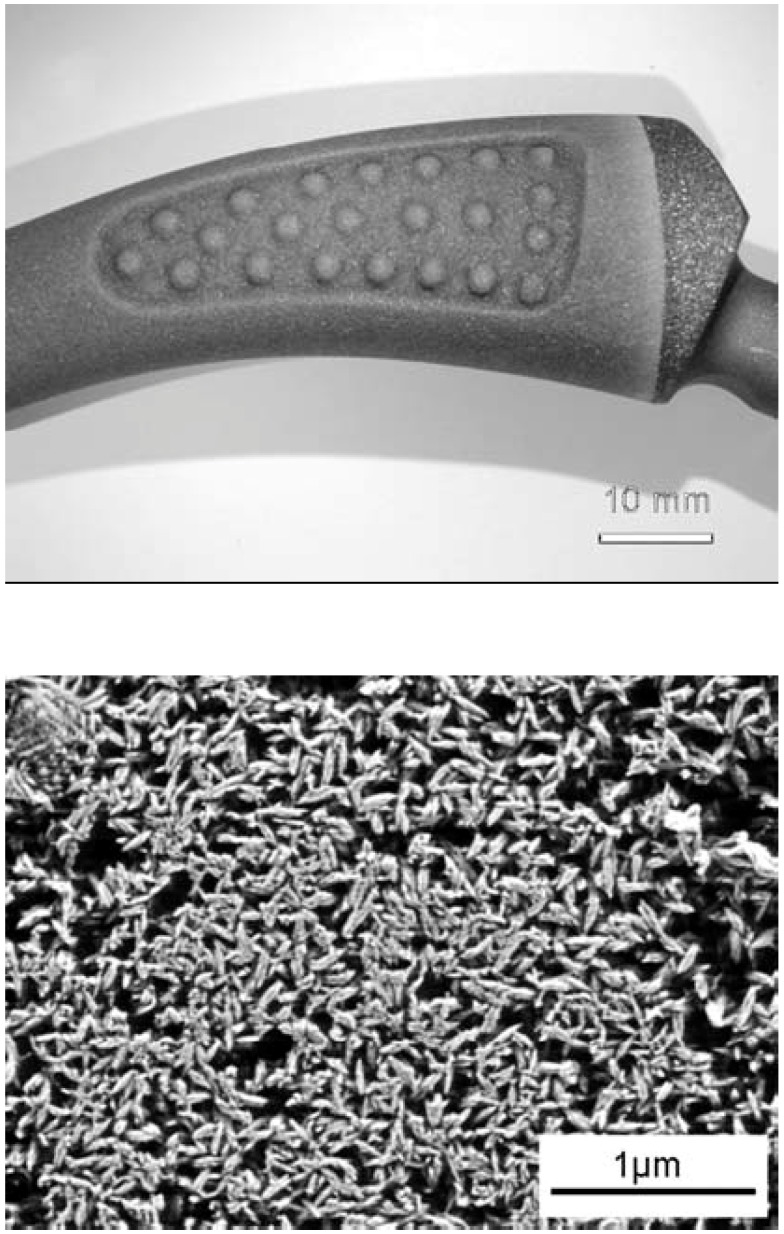
(a) A photo of a titanium implant coated with electrochemically deposited HA at 37 °C (Cenos^®^ BoneMaster); (b) A micrograph of a titanium implant surface coated with electrochemically deposited HA at 37 °C. Reprinted from Ref. [534] with permission. Other micrographs of nano-CDHA coatings biomimetically deposited on NaOH-treated Ti6Al4V surfaces might be found in Ref. [535].

Owing to their low mechanical properties, the use of calcium orthophosphates in load-bearing applications is rather limited: calcium orthophosphates are too stiff and brittle for such use. Today’s solutions for weight-bearing applications rely mostly on biologically friendly metals, like cobalt-chromium alloys, titanium and its alloys, as well as stainless steel 316L, but problems with stress-shielding and long-term service can cause failures. All these metals, although nontoxic, are always bioinert and cannot bond to bone directly. In order to improve the biological properties of the metallic implants, nanostructured calcium orthophosphates (mainly, apatites) are generally used as a coating material to accelerate bone growth and enhance bone fixation [173,174,280,351,455,511,512,513,514,515,516,517,518,519,520,521,522,523,524,525,526,527,528,529,530,531,532]. The coating techniques include thermal spraying, sputter coating, pulsed laser deposition, dynamic mixing method, dip coating, sol-gel method, electrophoretic deposition, biomimetic process, hot isostatic pressing and some other methods [533]. In the majority cases, the coatings are composed of uniform nanocrystalline apatites (Figure 7). They are capable in performing bone formation and promoting direct osseointegration with juxtaposed bone [536,537,538,539]. For example, an enhanced new bone formation can be clearly seen on nanophase HA-coated tantalum compared to micro-scale HA-coated tantalum and non-coated tantalum (see Figure 2 in Ref. [116]). Furthermore, nanostructured calcium orthophosphates might be used as a coating material to impart surface bioactivity to other materials, e.g., glasses [540] and polymers [541,542]. Finally but yet importantly, such coatings might be patterned, e.g., by laser direct writing [463] or electrohydrodynamic atomization spraying technique [543].

### 8.2. Nanodimensional and Nanocrystalline Calcium Orthophosphates and Bone-Related Cells

It is well accepted that bone-related cells (especially, osteoblasts and osteoclasts) play the key roles in the physiological formation of calcified tissues. Bone-related cells not only are speculated to take part in the formation of biominerals and macrostructure constructions of bones, but they also continuously modulate the density, regeneration and degradation of bones. Therefore, understanding the relationship between the bone-related cells and nanosized calcium orthophosphates has been paid much attention in order to elucidate the formation mechanism of bones, to prevent and cure bone-related diseases and to design novel biomaterials. Better structural biomimicity and osteoconductivity can be achieved using nanodimensional and nanocrystalline calcium orthophosphates [166,167,173,174,544,545,546]. Biocompatibility of such biomaterials is the key question for their application possibility for clinical use. For example, adhesion, proliferation and differentiation of mesenchymal stem cells were studied on nanosized HA/polyamide biocomposite scaffolds. The results indicated that such biocomposites exhibited a good biocompatibility and an extensive osteoconductivity with host bone in vitro and in vivo and proved that nanosized HA/polyamide scaffolds had a potential to be used in orthopedic, reconstructive and maxillofacial surgery [547,548,549].

Most results demonstrate that nanostructured HA can improve cell attachment and mineralization in vivo, which suggests that nanosized HA may be a better candidate for clinical use in terms of bioactivity [173,174,178,550,551,552]. The size effects of nanodimensional HA on bone-related cells, as well as the influence of crystallinity of nanosized HA were studied [409,553]. Different HA nanoparticles, typically of 20 ± 5, 40 ± 10 and 80 ± 12 nm in diameter, were prepared and their effects on the proliferation of two types of bone-related cells, bone marrow mesenchymal stem cells (MSCs) and osteosarcoma cells (U2OS and MG63) were studied. The cell culture experiments showed an improved cytophilicity of the nanophase HA if compared to the submicron-sized HA. A greater cell viability and proliferation of MSCs were measured for nanosized HA, remarkably for 20 nm-sized particles. However, the opposite phenomenon occurred for bone tumour cells when HA nanoparticles were co-cultured with cells. HA nanoparticles can inhibit proliferation of U2OS and MG63 cells and the inhibited strengths were inversely proportion to the particle size, i.e. smaller nanoparticles possessed a greater ability to prevent cell proliferation. This suggests that HA nanoparticles can exhibit favorable cell proliferation to optimize biological functionality, in which the particle dimensions are believed to play a key role. These in vitro findings are of a great significance for the understanding of cytophilicity and biological activity of nanoparticles during biomineralization [409].

Studies confirmed that nanosized ACP had an improved bioactivity if compared to nanosized HA since a better adhesion and proliferation of osteogenic cells had been observed on the ACP substrates [554]. However, in order to understand the influence of crystallinity of the nanosized calcium orthophosphates on the osteogenic cells correctly, it was critical to use ACP and HA nanoparticles of the same size distribution [553]. ACP and HA particles of ~20 nm size were synthesized and the effects of crystallinity were studied. The adhesion, proliferation and differentiation of MSC cells were measured on both ACP and HA films and compared at the same size scale. Surprisingly, more cells were adsorbed and proliferated on the films of the well-crystallized nanosized HA than those on the nano-ACP films. Alkaline phosphatase activity assay and RT-PCR assay were also used to evaluate the differentiation of MSC cells. The results showed that the differentiation of MSC cells from osteoblasts was promoted significantly by nanosized HA. These experimental phenomena clearly demonstrate that the crystallized phase of HA provides a better substrate for MSC cells than ACP, when the factor of size effect is removed. This new view on the relationship between the crystallinity of calcium orthophosphates and the responses of MSCs emphasized the importance of both size and phase control in the application of biomedical materials [553,554].

Cells are sufficiently sensitive and nanoscale alterations in topography might elicit diverse cell behavior [555,556,557]. How cells can recognize the particle size and other very small differences in the properties of nanosized HA in these experiments remains unclear. Actually, determining the mechanisms whereby calcium orthophosphate nanoparticles and their sizes exert effects on bone-related cells will require further systematic studies.

### 8.3. Dental Applications

Dental caries is a ubiquitous and worldwide oral disease. At the initial stage of caries lesions, bacteria cause damage of dental enamel, which is the exterior coating of teeth and possesses remarkable hardness and resistance. As the most highly mineralized structure in vertebrate bodies, enamel is composed of numerous needle-like apatite nanocrystals, which are bundled in parallel ordered prisms to ensure unique mechanical strength and biological protection (see section 5.2 above). As a non-living tissue, the main constituent (~97 wt.%) of mature enamel is inorganic nanodimensional apatite so that enamel is scarcely self-repaired by living organisms after substantial mineral loss. Filling with artificial materials is a conventional treatment to repair damaged enamel. However, secondary caries frequently arise at the interfaces between the tooth and foreign materials [558].

Nanodimensional HA and CDHA are often considered as model compounds of dental enamel due to the chemical and phase similarities [88,89,99]. Therefore, the remineralization of enamel minerals by using nanodimensional apatite or other calcium orthophosphates is suggested in dental research. For example, nanodimentional apatite-containing toothpastes could promote a partial remineralization of demineralized enamel [559,560,561], as well as possess some whitening effect [562]. Furthermore, nanosized HA might be added to a fluoride-containing mouthrinse [563]. A remineralization potential of sports drink, containing nanosized HA, was also investigated [564]. A positive influence of addition of nanodimensional β-TCP against acid demineralization and promoted remineralization of enamel surface was detected as well [565]. Unfortunately, these chemically analogous compounds of enamel are not widely applied in clinical practices. The native structure of dental enamel is too complex to be remodeled and the synthesized apatite crystallites often have different dimensions, morphologies and orientations from the natural ones, which result in a poor adhesion and mechanical strength during dental restoration. Recent advances in biomineralization also indicate that features of smaller HA nanoparticles may more closely approximate features of biological apatite than features of the larger HA particles that are conventionally used [13]. For example, it has been demonstrated that HA nanoparticles can be self-assembled to form enamel-like structures in the laboratory [204]. Therefore, a biomimetic technique is suggested as follows: the localized repair of the enamel surface can be improved by the HA nanoparticles (dimension of ~20 nm), analogues to the basic building blocks of enamel rods. Furthermore, it is found that nanosized HA can adsorb onto the enamel surface strongly and can even be integrated into the natural enamel structure [566].

It is surprising but ~20 nm HA nanoparticles can inhibit significantly a mineral loss from the enamel surface [207]. Without any treatment, the demineralization of the natural enamel surface was remarkable in acidic solution (pH ~4.5 ± 0.1, experimental period of 2 days) and damaged sites were observed. The mass loss rate was about 0.12 ± 0.04 mg/mm^2^ per day. In contrast, a layer of nanosized HA on the treated enamel surface was almost unchanged in acidic solution. The rate of mass loss of nanosized HA-coated enamel approached zero (<0.02 mg/mm^2^ per day), which was beyond the sensitivity of the detection methods. Since the coating by nanosized HA appeared to be insensitive to dissolution, the underlying enamel surface was well protected under slightly acidic conditions. Furthermore, the 20 nm HA-coated enamel surface had a hardness of 4.6 ± 0.4 GPa and an elastic modulus of 95.6 ± 8.4 GPa. These data appeared to be very similar to those of natural enamel samples, which are 4.2 ± 0.2 and 94.1 ± 5.4 GPa, respectively [207].

The similarity of ~20 nm-sized HA and building blocks of biological apatite of dental enamel results in a good fixation of artificial biomaterials to natural tissues. Moreover, the enamel structure is even reinforced by nanosized HA since secondary caries formation is suppressed and hardness is retained [558,567,568]. This strategy may have prospective applications in dentistry as it offers an easy but effective method to reconstruct tooth enamel that is suffering from mineral losses. Generally, these studies also suggest that analogues of nano-building blocks of biominerals should be highlighted in the entire subject of biomineralization.

In the case of nanodimensional DCPA, decreasing of DCPA particle dimensions were found to increase the Ca- and PO_4_-ions release from DCPA-based biocomposites. Therefore, nano-DCPA-based biocomposites, possessing both a high strength and good release of Ca- and PO_4_-ions, may provide the needed and unique combination of stress-bearing and caries-inhibiting capabilities suitable for dental applications [441].

### 8.4. Other Applications

Several other applications of nanodimensional and nanostructured calcium orthophosphates are in progress, some of which are described here. Surface modification of calcium orthophosphate nanoparticles was performed in order to modulate their colloid stability, prevent dissolution in the case of low pH, avoid inflammation, serve as an intermediate layer to allow strong bond formation between HA/polymer matrices and potentially enhance its bioactivity or improves its conjugation ability with special functional groups [12,569,570,571,572]. In another aspect, calcium orthophosphate nanoparticles have also served as non-viral carriers for drug delivery and gene therapy due to their established biocompatibility, ease of handling and notorious adsorption affinity [187,259,281,412,438,453,573,574,575,576,577,578,579,580,581,582]. Furthermore, they can be stably loaded with radioisotopes [281]. After loading with genes or drugs by adsorption, nanodimensional apatites provide a protective environment that shields them from degradation while providing a convenient pathway for cell membrane penetration and controlled release of the genes or drugs [413]. The experimental results proved that nanodimensional calcium orthophosphates possessed a higher penetration rate into cell membranes and their transfection efficiency could be 25-fold higher than that of the micron-sized particles. Furthermore, due to the larger specific surface areas, nanodimensional calcium orthophosphates can hold larger load amounts of drugs than coarser particles. These results indicate the potential of nanosized calcium orthophosphates in gene delivery and as drug carriers [413,583,584,585,586].

A transfer of functional foreign nucleic acids (DNA or RNA) into nuclei of living cells (transfection) with the aim of repairing missing cell function and to provide means to enhance or silence gene expression is currently used extensively in the laboratory and is fast becoming a therapeutic reality. As nucleic acids alone are unable to penetrate the cell wall, efficient carriers are required [587,588]. Calcium orthophosphate nanoparticles can be represented as a unique class of the non-viral vectors, which can serve as efficient and alternative DNA carriers for targeted delivery of genes [259,577,589,590,591,592,593,594,595,596,597,598,599,600] and cells [456,601,602,603,604,605,606]. Interestingly, but the transfection efficiency of calcium orthophosphates were found to depend on Ca/P ionic ratio: namely, calcium orthophosphates with Ca/P = 1.30 ratio exhibited a fourfold increase in the transfection efficiency over the ones with Ca/P = 1.65 ratio composition [259]. This data emphasizes the importance of understanding the interaction between calcium orthophosphates and DNA to optimize the DNA uptake and its channeling to the nucleus of the cell. Besides, it has been demonstrated that surface modified calcium orthophosphate nanoparticles can be used *in vivo* to target genes specifically to a liver [607]. Attachment of galactose moiety onto the particle surface has increased the targetability of calcium orthophosphate nanoparticles. Furthermore, this surface modification makes it possible for site-specific gene delivery [607]. Block-copolymer/calcium orthophosphate nanoparticle assemblies were prepared and used for cell transfection; a high biocompatibility of this system was emphasized [608]. Furthermore, vaccination to protect against human infectious diseases may be enhanced by using adjuvants that can selectively stimulate immunoregulatory responses and calcium orthophosphate nanoparticles were found to be suitable for such purposes [609,610].

In all these new applications of calcium orthophosphate nanoparticles, knowledge of the exact internalization pathway into the cells represents the first necessary step towards the detailed investigation and optimization of the functional mechanism. The main groups of pathways into the cell are diffusion, passive and active transport, as well as a number of endocytic mechanisms [579]. Bigger particles of far above 10 nm are internalized by eukaryotic cells through the endocytic pathways including phagocytosis, macropinocytosis, clathrin-mediated endocytosis and non-clathrin-mediated endocytosis such as internalization via caveolae. To date, the exact internalization pathway of calcium orthophosphate nanoparticles into cells has not been determined and there are many questions that remain to be answered, particularly, concerning possible interactions of calcium orthophosphates with nucleic acids. Furthermore, the mechanisms of cellular uptake and transport to the cell nucleus of calcium orthophosphate/DNA complexes remain unclear either. Therefore, there is a need to conduct a focused study on the synthesis of various forms of nanostructured calcium orthophosphates that could elucidate the mechanisms of binding, transport and release of attached plasmid DNA for understanding the gene delivery method. Research is also warranted to understand the tracking of DNA intracellularly [603] to understand the release and transport of DNA into cellular nuclei. Already, some data are available that clathrin-mediated endocytosis might be responsible for the uptake of HA nanoparticles [579].

Concerning the healing abilities of calcium orthophosphate nanoparticles, an *in vitro* inhibiting effect and even apoptotic action of un-functionalized HA nanoparticles of about 50 nm diameter on a hepatoma cell line in the concentration range of 50–200 mg/1 was reported [611]. A similar inhibiting effect was discovered for discrete HA nanoparticles, which appeared to cause apoptosis of leukemia P388 cells [119] and rat macrophages [612]. This effect might be due to a harmful increase in the intracellular calcium concentration.

Hollow nanospheres are extremely attractive constructions because they can greatly enhance the load quantity. Though these novel biomaterials can improve the total intake of drugs, they also bring new problems, e.g., uncontrolled release kinetics and unreasonable metabolism pathway of the carriers [613]. In order to solve these problems, calcium orthophosphates were selected as nanospherical carriers [437,438,581,614,615]. The most important feature is that the hollow-structured calcium orthophosphate nanospheres can be collapsed and transferred into pin-shaped crystallites under ultrasonic treatment. During this transformation, the encapsulated drugs and chemicals are released [453,616].

Hollow calcium orthophosphate nanospheres with sizes ranged from 110 to 180 nm were synthesized by an ultrasonic-assisted wet chemical reaction in the presence of a modifier [616]. In addition, such nanospheres might be prepared through nanoemulsions [617]. Transmission electron microscopy investigations revealed that the uniform calcium orthophosphate nanospheres were formed and they were well dispersed in the solutions. Thickness of the shells was about 45 nm; thus, the nanospheres always had ~60 nm-sized internal cavities, which could be used to load drugs. The hollow nanospheres appeared to be stable in both air and aqueous solutions without ultrasonic application. However, when an ultrasonic treatment (40 kHz, 150 W) was applied, the hollow structures deconstructed to form pin-like nanocrystals of calcium orthophosphates [616]. Different from a free and slow diffusion of encapsulated drugs from the cavity through the shells [184], the release kinetics in this system was triggered and controlled by ultrasound. Furthermore, the power density of ultrasound can conveniently regulate the release dynamics. Besides, the formed pin-like calcium orthophosphate nanocrystals had similar behavior to the biological apatite of bones. Thus, a combination of the hollow calcium orthophosphate nanospheres and ultrasonic treatment might provide a good system for drug delivery and release [616].

Interestingly (although this is beyond the subject of biomaterials), but calcium orthophosphate nanoparticles with a mean size of 150 ± 20 nm filled with a solution containing luminol, haematin and fluorescein were found to improve the ease and accuracy of H_2_O_2_ sensing [618].

## 9. Summary and Perspectives

As the basic building blocks of calcified tissues of mammals, poorly crystalline calcium orthophosphate nanoparticles of the apatitic structure play an important role in the construction of these biominerals. Therefore, they appear to be almost the ideal biomaterials due to their good biocompatibility and bioresorbability. Even more enhanced applications are expected in drug delivery systems [619]. However, there is still an unanswered question concerning their structure: whether nanodimensional apatites appear to be almost amorphous (according to numerous results of X-ray diffraction studies) due to their nanodimensions of well-crystallized structures or due to a really amorphous (*i.e*., retaining only a short-range order at the scale of few atomic neighbors) matter? A good attempt to discuss this topic is available in literature [620], where the interested readers are referred to.

In future, an ability to functionalize surfaces with different molecules of varying nature and dimensions by means of their attachment to cells will enable them to act selectively on biological species such as proteins and peptides. The capability of synthesizing and processing of nanodimensional and nanocrystalline calcium orthophosphates with the controlled structures and topographies, in attempts to simulate the basic nano-units of bones and teeth, will provide a possibility of designing novel proactive bioceramics necessary for enhanced repair efficacy. The various primary positive results on the biocompatibility and biomimicity of novel nanostructured bioceramics merit further confirmations.

Much work remains to be undertaken to address the following key challenges and critical issues of nanodimensional and nanocrystalline calcium orthophosphates [621]:

- Consistency of the processing technologies;
- Optimization the structure and properties mimicking bones;
- Matching the strength of nanodimensional and nanocrystalline constructs with those of bones in order to provide a uniform distribution of stresses (load sharing);
- Optimizing bioresorption without comprising the mechanical properties;
- Assessing the inflammatory response to validate their biosafety.

Furthermore, substantial research efforts are required in the analysis of cells and their different behaviors with regard to their interactions with nanodimensional and nanocrystalline calcium orthophosphates [621]. An important but still unsolved question is how the cells can recognize the particle dimensions and crystallinity of nanosized calcium orthophosphates. What is the signal for nanodimensional biomaterials to promote cell proliferation and differentiation and how can the pathways be found out? Presumably, nanoparticles with smaller sizes can enter into cells more readily but this suggestion needs to be confirmed experimentally. Namely, the pathways for the nanoparticles to enter the cells through the membranes should be revealed [622]. A greater influence of the hydrated surface layer with labile ionic species of smaller particles and crystals (see section 6 for the details) might be another possible option, to be confirmed experimentally as well. Then, it is important to examine the metabolism process of calcium orthophosphate nanoparticles inside cells, so the existing forms of these particles during the biological processes could be understood. Further, a critical step will be the investigation of possible changes of gene or protein expression in the absence and presence of various nanoparticles of calcium orthophosphates, which may directly be related to cell proliferation and differentiation [13].

An understanding of the interaction among nanoparticles and living cells is still a great challenge [621]. Future studies will focus on (1) the detailed interfacial structure of nanodimensional calcium orthophosphates and the specific adsorption of proteins or other matrices; (2) uptake processes of the nanoparticles by cells; (3) metabolism of calcium orthophosphates nanoparticles inside cells and its interference with physiological reactions. Another important topic is a biological security of nanoparticles in general [134,135,623,624] and those of calcium orthophosphates particularly [625]. For example, toxicity of HA nanoparticles was found to vary considerably, which was related to their physico-chemical properties. Cell death correlated strongly with nanoparticle load. The intracellular dissolution of HA nanoparticles as a function of time suggests that increased cytoplasmic calcium load is likely to be the cause of cell death [625]. Although nanodimensional calcium orthophosphates promote bone repair, these nanoparticles also penetrate biological organisms more readily, e.g., to enter the circulatory system by penetration into blood vessels. Thus, understanding the biological influence of nanosized and nanocrystalline calcium orthophosphates is essential for a future development of bionanotechnology [626]. This interdisciplinary approach is very complicated and the effective collaboration of scientists from different disciplines is the key [13].

## 10. Conclusions

With a high surface area, un-agglomerated nanodimensional and nanocrystalline bioceramic particles are of interest for many applications including injectable or controlled setting bone cements, high strength porous or non-porous synthetic bone grafts and the reinforcing phase in nanocomposites that attempt to mimic the complex structure and superior mechanical properties of bone. Therefore, nanosized and nanocrystalline calcium orthophosphates have already gained much regard in the biomedical field due to their superior biocompatibility and biomechanical properties. This is easily seen from a permanent increasing of the amount of publications. At present, apatites (HA and CDHA) and β-TCP are the major calcium orthophosphates used in clinics. Currently, nanodimensional apatites are used primarily as bioactive coatings on bioinert materials like titanium and its alloys, in bone tissue repairs and implants, as well as for drug delivery purposes. The nanosized β-TCP exhibits a significant biological affinity and activity and responds very well to the physiological environment. A lot of research is expected for much enhanced applications of the nanodimensional and nanocrystalline calcium orthophosphates for both drug delivery systems and as resorbable scaffolds that can be replaced by the endogenous hard tissues with the passage of time [137,628].

Although the nanostructured biomaterials may have many potential advantages in the context of promoting bone cell responses [431,432,433,557], it is important to remember that studies on nanophase materials have only just begun; there are still many other issues regarding human health that must be answered. A rapid technical development of nanometer-scaled particles in the biomedical field leads to concerns regarding the unknown risks of such materials since particles of very low size have higher reactivity and effectiveness [623,624]. These nanoparticles might induce inflammatory reactions, cytotoxicity, oxidative stresses or thrombogenesis when injected for drug delivery purposes. Namely, small nanoparticles may enter the human body through pores and may accumulate in the cells of the respiratory or other organ systems (when becoming dislodged through wear debris) and the health effects are yet to be largely known. This could happen during commercial-scale processing of the nanoparticles as well as using these materials as implants [629]. Nanoparticles might be the objects whose existence has not been assumed by living body defense system [18,134,135]. Up to now, only a small number of short-term and small-scale health effects of single nanomaterials have been examined in toxicological studies, usually of the lungs [624]. Therefore, prior to clinical applications, any toxicity concerns of the nanophase materials [630,631,632,633,634,635] need to be overcome.

In summary, despite the challenges that lie ahead, significant evidence now exists elucidating that nanophase biomaterials represent an important growing area of research that may improve bonding between the implants and the surrounding tissues. It has proven to be a versatile approach that can increase bone cell functions on a wide range of orthopedic implant chemistries. Even if the nanodimensional and nanocrystalline calcium orthophosphates do not provide the ultimate answer for increasing bone cell responses (due to some potential problems as mentioned above), researchers have learned a tremendous amount of information concerning bone cell recognition with nanostructured surfaces that will most certainly aid in improving orthopedic implant efficacy [134,135].

## 11. Post-Conclusion Remarks

According to Prof. D. F. Williams, the term “nanomaterial” should not exist because it is senseless [108]. Following this logic, the term “nanoapatite” is senseless as well. However, it is presented in the titles of several publications, namely Refs. [491,513,537,550]. In a slightly modified form, the term “nano-apatite” is presented in the titles of several other publications, namely Refs. [74,83,205,412,515,596,628]. Furthermore, similar terms “nano-HA” [86,464,493,543,560,567], “nano-hydroxyapatite” [40,72,75,76,79,80,85,119,178,179,264,265,278,307,383,389,467,472,491,511,512,525,547,549,556,561,562,563,568,570,572] and “nanohydroxyapatite” [71,77,82,151,186,218,219,221,231,246,283,465,540,547,615] are presented in the titles of still other publications. Presumably, it is wiser not to use these terms anymore.
