# Expression of Concern: Nanodimensional and Nanocrystalline Apatites and Other Calcium Orthophosphates in Biomedical Engineering, Biology and Medicine. *Materials* 2009, *2*, 1975–2045

**DOI:** 10.3390/ma9090752

**Published:** 2016-09-02

**Authors:** Materials Editorial Office, Maryam Tabrizian

**Affiliations:** 1 St. Alban-Anlage 66, 4052 Basel, Switzerland; 2 Department of Biomedical Engineering, Faculty of Medicine/Faculty of Dentistry, Duff Medical Science Building, Room 313, 3775 University Street, Montreal, QC H3A 2B4, Canada; maryam.tabrizian@mcgill.ca

We wish to make readers aware that the text in the published paper [1] contains substantial overlap with a previous paper by the same author [2]. Since [1] is an expanded version of [2] and the authorship is identical, we have taken the decision that it should remain in the literature. However, readers should be aware that the two papers are identical in many aspects. We regret that this issue was not detected prior to publication and apologize to readers of *Materials* for any inconvenience.
